# Dataset of 3D computer models of Late Miocene Mount Messenger Formation outcrops in New Zealand, built with UAV drones

**DOI:** 10.1016/j.dib.2024.110035

**Published:** 2024-01-09

**Authors:** Erman H. Kamaruzaman, Andrew D. La Croix, Peter J.J. Kamp

**Affiliations:** Sedimentary Environments and Analogues Research Group, Earth and Environmental Sciences, School of Science, University of Waikato, Private Bag 3105, Hamilton 3240, New Zealand

**Keywords:** Taranaki Basin, Late Miocene, Virtual fieldwork, Deep-water sedimentary systems, Mount Messenger Formation

## Abstract

The aim of constructing 3D computer models of outcrops of the Mount Messenger Formation using unmanned aerial vehicle (UAV) drone technology was to enable better visualization and potential for analysis of deep-water sedimentary systems in Taranaki Basin, New Zealand. The Late Miocene-aged strata crop out along the north Taranaki coast of western North Island, New Zealand. The Mount Messenger Formation sandstone and siltstone beds are outstanding examples of deep-water sedimentary strata. These strata can be observed in outcrop sections, as well as in offshore drillholes (wireline logs) and in seismic reflection data acquired immediately offshore of the north Taranaki coastal section. In previous research undertaken on the Mount Messenger Formation in North Taranaki Basin, geologists used photographs and coupled these with observations and descriptions of strata in the field. Modern UAV drone technology now enables 3D perspectives to be obtained of outcrop sections, which greatly improves geometrical analysis of the rocks. This type of analysis, coupled with mapping of seismic reflection data in the immediate offshore area has enabled us to better understand the nature of Mount Messenger Formation deep-water sedimentary strata and to interpret the associated paleogeography with implications for energy resource exploration and evaluation.

Using UAV drone photogrammetry, we acquired ∼3000 images of the Mount Messenger Formation outcrop at four locations along the north Taranaki coast. Drone surveys were conducted using a real-time kinetic (RTK) global positioning system (GPS) for accurate geolocation. The surveys were conducted on a DJI Phantom 4 drone, with a focal length of 24 mm with a 20-megapixel resolution. Survey images overlapped by 80–90%. The drone work adhered to the rules and regulations of the Aviation Security Service and the University of Waikato, New Zealand. Images were captured using programmed flight paths where the drone faced the outcrops at distances ranging from ∼3–7 m.

3D computer models were constructed using Pix4Dmapper version 4.4.12 to generate dense 3D point clouds, digital surface models (DSMs), triangle meshes, and orthomosaic images of the outcrops (i.e., 3D models). Once the 3D computer models of the outcrops were constructed, they were exported out of Pix4Dmapper as ArcGIS Scene Layer Package format (.slpk) and loaded into ArcGIS Pro version 3.0.3 for further analysis.

The 3D computer models comprise a rich and valuable scientific dataset that can enhance geological analysis of sedimentary strata beyond the capabilities of photographs and manual fieldwork. These models allow desktop analysis of the geology and “virtual fieldwork” by imaging areas that are commonly inaccessible on foot due to their high elevation above ground level, location in rugged and steep terrane, as well as periodic intertidal flooding. This electronic geological dataset is stored in commonly used spatial format and plain-text ASCII files, allowing the preservation of geological data in digital records, especially when the outcrops are prone to erosion and cover by vegetation. The drone model dataset can be reused by the scientific community for virtual geological fieldwork, as petroleum and water reservoir analogues, as well as for research on coastal, environmental and geotechnical topics.

Specifications Table

**Table:** 

Subject	Earth and Planetary Sciences / Geology
Specific subject area	Construction of 3D computer model dataset based on geolocated UAV drone images of Mount Messenger Formation outcrops on coastal sections of Taranaki Basin, North Island, New Zealand
Data format	Raw
Type of data	.slpk files (Scene Layer Packages) .xyz files (ASCII data with X,Y and Z coordinates) .ppkx files (ArcGIS Pro Project Packages)
Data collection	Approximately three thousand photographs were captured using UAV drones at four geological outcrop locations along the North Taranaki coast, North Island, New Zealand (Figs. 1 and 2). Images were captured using a DJI Phantom 4 drone connected to a real-time kinetic (RTK) global positioning system (GPS)
	for accurate geolocation. In addition to the RTK GPS, we also used a Leica GPS system for precise ground geolocation. Drone surveys of the four sites used programmed flight paths operating on the WGS 84 coordinate reference system and EGM 2008 Geoid. The focal length of the drone camera was 24 mm with a 20-megapixel resolution. The images captured outcrop at an average of 3–10 cm Ground Sampling Distance (GSD). Survey images were overlapped by 80–90%. Once the surveys were completed, drone images were processed using Pix4Dmapper version 4.4.12 to generate dense 3D point clouds, digital surface models (DSMs), triangle meshes, and orthomosaic images of the outcrops (i.e., 3D models). Once 3D outcrop models were built, they were exported out of Pix4Dmapper in Scene Layer Package format, and then imported into ArcGIS Pro-version 3.0.3. Drone surveys were conducted on relatively fair-weather days where cloud cover was minimal and with wind speeds below 32 kms per hour to ensure sufficient exposure on the outcrops for good quality photographs. Permission from the Department of Conservation (New Zealand Government) and a private landowner were obtained prior to conducting the surveys. Licensed drone operators were in charge of surveys, which were flown on the 16th- 17th of June, 12th of August, and 28th-29th of October 2023.
Data source location	The location of the sites where drone surveys were conducted are as follow: 1. Site 1 - Latitude: 38°39′56.49″S, Longitude: 174°37′48.01″E 2. Site 2 - Latitude: 38°42′27.39″S, Longitude: 174°37′1.52″E 3. Site 3 - Latitude: 38°46′9.60″S, Longitude: 174°36′0.00″E 4. Site 4 - Latitude: 38°49′0.24″S, Longitude: 174°35′9.04″E These locations are situated on the west coast of New Zealand's North Island (Fig. 1, Fig. 2).
Data accessibility	Repository name: Harvard Dataverse 2023 Data identification number: https://doi.org/10.7910/DVN/I0C6 × 3 Direct URL to data: 3D drone outcrop models of the Mount Messenger Formation, New Zealand - Harvard Dataverse Instructions for accessing these data: Log in to the database using your credentials and download the data.
Related research article	

## 1 Value of the Data

• This dataset is valuable because it can be used to enhance visualization of large-scale geological layers. The 3D models provide a more precise and detailed representation of geological strata compared with photographs and hand-drawn sketches, which are historically how geological field observations have been recorded. This enhanced vizualisation helps geologists, geophysicists and engineers better understand the stratigraphic and structural characteristics of strata in outcrops that were deposited in ancient deep-water sedimentary environments.
• The value of the data also lies in its capacity to facilitate virtual fieldwork or field trips. The 3D models enable researchers and students to conduct digital field analysis of the geological layers. Many sections of the Mount Messenger Formation outcrop are inaccessible due to their location at high elevation above ground level, occurrence in rugged and steep terrane, as well as coastal areas that may be periodically submerged during high tides. Moreover, UAV drone surveys require permission from landowners, which may limit access to field locations. The 3D models of rock outcrops are of particular use for scientists and students conducting research when travel is not permitted (e.g., during the COVID-19 lockdown), or if they are physically unable to undertake fieldwork.
• This dataset is valuable because it digitally preserves geological data and observations. The 3D computer models are digital records of the geological outcrops and preserve this for use indefinitely into the future. This is particularly important for the Mount Messenger Formation outcrops along the west coast of the North Island of New Zealand because they are prone to coastal erosion and significant vegetation cover.
• This dataset can be used by other scientists and students for research and education relating to sedimentology, stratigraphy, and sedimentary basin analysis of paleo deep-water sedimentary systems.
• This dataset can be used by researchers and industry practitioners for flow modelling of aquifers, petroleum reservoirs, and carbon capture and geostorage intervals. The 3D computer models are analogues to other reservoir systems globally that were deposited by deep-water sedimentary systems and can be used to estimate flow properties such as porosity and permeability distributions.
• This dataset can be used for coastal geological and geomorphological research. The models will help us understand how coastal erosion develops over time. For instance, outcrops at Sites 3 and 4 are situated along the coast and are subject to constant erosion from marine processes (Fig. 2C and D). Throughout our multiple visits to the sites to conduct drone surveys, we noticed parts of the outcrops had collapsed and eroded between visits. Apart from coastal studies, this dataset can be used by other researchers for environmental and geotechnical analysis. The outcrop models can assist researchers in studies of landslides. For example, the outcrop at Site 2 (Fig. 2B) is adjacent to State Highway 3 (i.e., Mokau Road). Landslides and rock falls are common in this area and cause serious environmental and safety issues and road closures.

## 2 Background

**Fig. 1:**
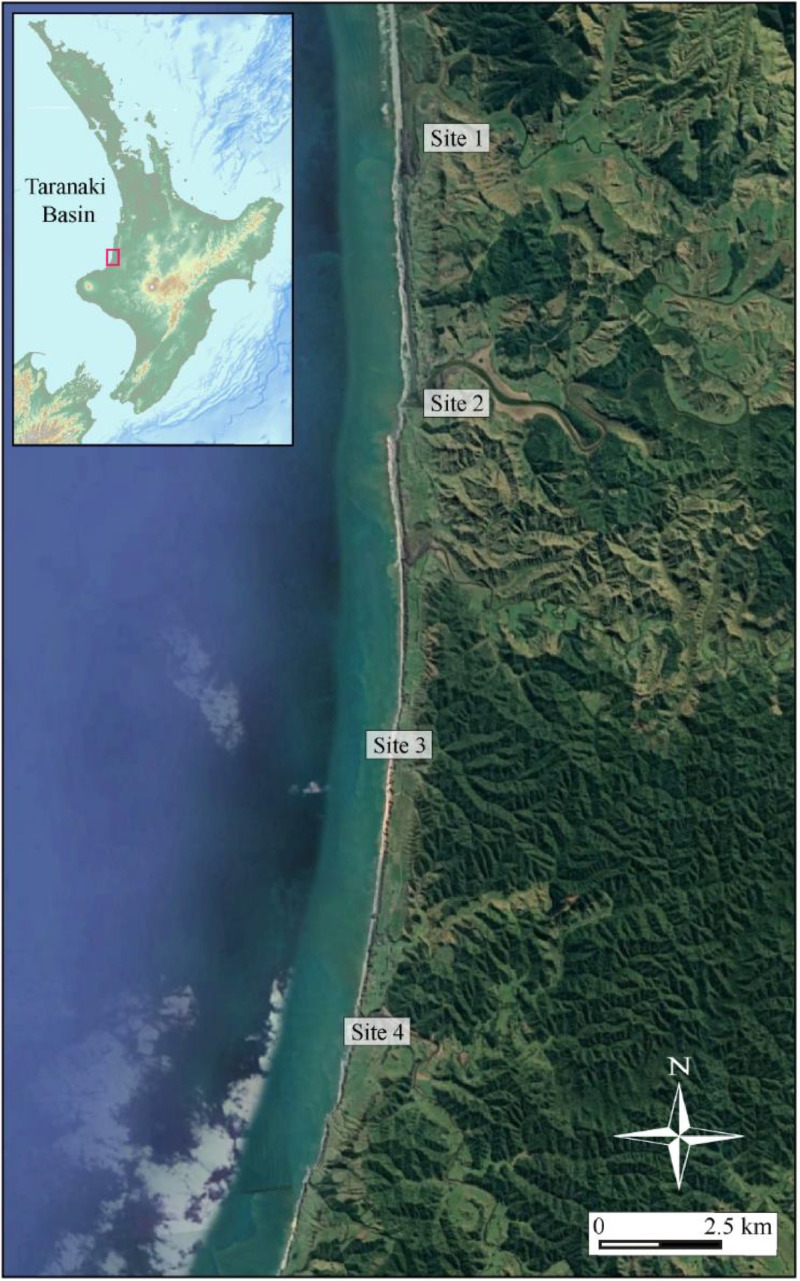
Overview map of the drone survey areas. The inset map shows the locations along the north Taranaki Coast, North Island, New Zealand (red polygon). (For interpretation of the references to color in this figure legend, the reader is referred to the web version of this article.)

**Fig. 2:**
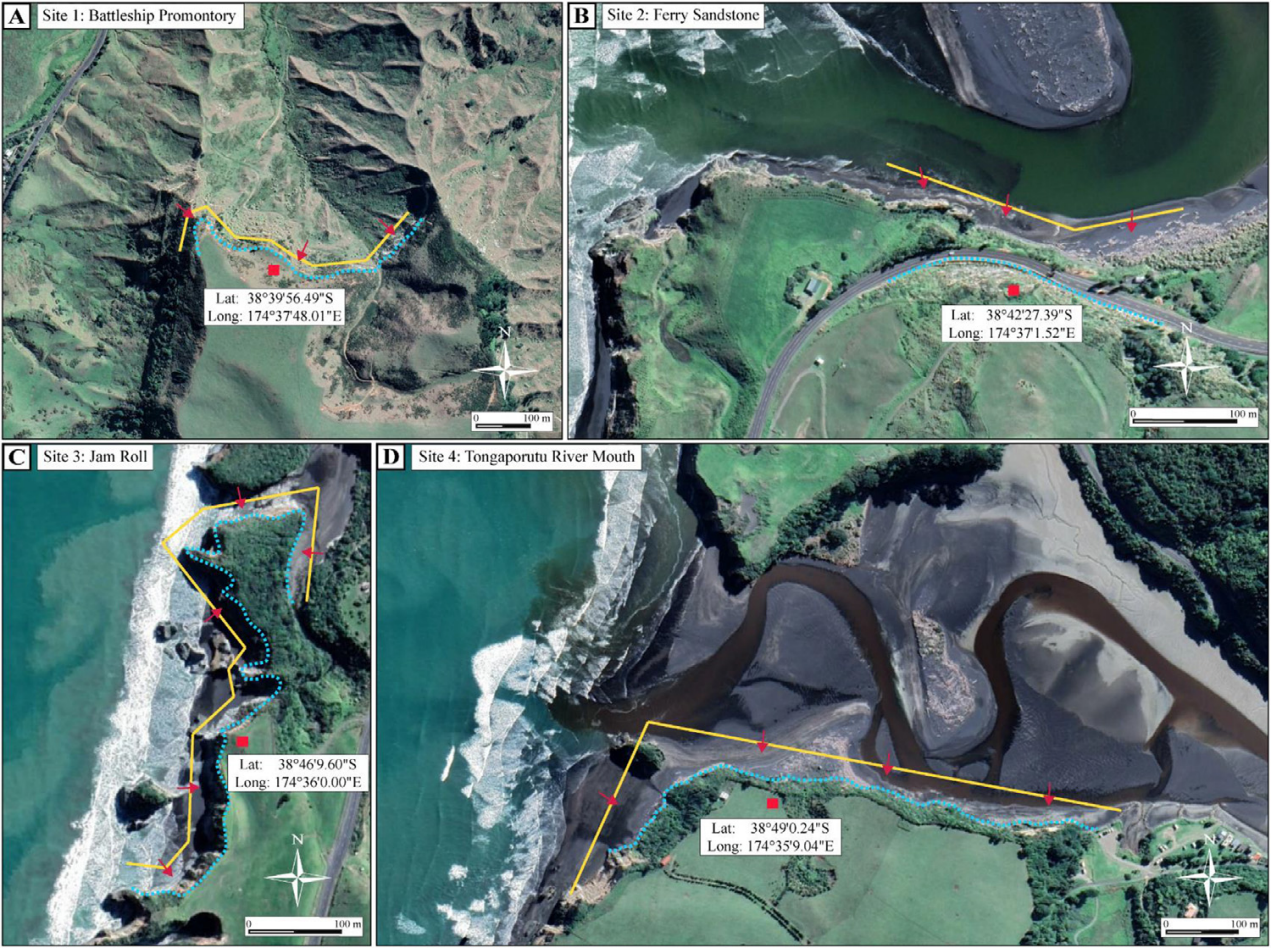
Close-up of the study areas: (A) Site 1 Battleship Promontory, (B) Site 2 Ferry Sandstone, (C) Site 3 Jam Roll and (D) Site 4 Tongaporutu River Mouth. The yellow lines are the drone flight paths, and the red arrows represent the direction that drone cameras were facing. (For interpretation of the references to color in this figure legend, the reader is referred to the web version of this article.)

The aim of constructing 3D computer models of geological outcrops of the Mount Messenger Formation was to better understand ancient deep-water sedimentary systems in Taranaki Basin. The Late Miocene-aged sedimentary strata that outcrop along the north Taranaki coast (Figs. 1 and 2) are excellent examples of deep-water sandstone and siltstone layers that are also present in offshore drill hole materials are imaged in seismic reflection data [1,4]. While previous studies [2,6] of these outcrops mainly used photographs, this study is the first to introduce UAV drones imagery to capture their sedimentary characteristics. Incorporating drones in this study allowed a broader coverage of areas than previously possible, including areas inaccessible by foot. Therefore, geological analysis of these deep-water sedimentary systems is improved, especially when viewed from a 3D perspective. This analysis yields a more holistic understanding of the ancient sedimentary system in the region, surpassing the insights provided by earlier studies. The drone images were processed in Pix4Dmapper version 4.4.12 with precise geolocation to generate the 3D computer models. Then, qualitative and quantitative stratigraphic analyses of the outcrops were carried out using ArcGIS Pro version 3.0.3 [5].

## 3 Data Description

The 3D computer models of rock outcrops along the north Taranaki coast, New Zealand, were built using UAV drones to capture images of the sedimentary characteristics of the Late Miocene Mount Messenger Formation beds. Each 3D computer model allows the geometric analysis of sandstone and siltstone layers to be calculated, including bed thickness, bed orientation, as well as stratal stacking patterns (e.g., comfortable and erosional) and related structural deformation (e.g., faulting and folding). The present-day elevation, aerial extent, length and camera-facing direction of the outcrops are displayed in Table 1. The dataset are stored in a public research database: doi:10.7910/DVN/I0C6 × 3 [3]

**Table 1:** Outcrop site elevation ranges and estimated length and aerial extent.

Site	Elevation range (mean sea level)	∼ Length	∼ Aerial extent
Site 1	145–210 m	900 m	0.20 km^2^
Site 2	7–80 m	370 m	0.035 km^2^
Site 3	0.5–22 m	800 m	0.092 km^2^
Site 4	2 to 25 m	900 m	0.052 km^2^

Seven 3D computer models of geological outcrops are stored in ArcGIS Scene Layer Package format (i.e., Site 1 Battleship Promontory.slpk, Site 2 Ferry Sandstone.slpk, Site 3 Jam Roll Part 1.slpk, Site 3 Jam Roll Part 2.slpk, Site 4 Tongaporutu River Mouth Part 1.slpk and Site 4 Tongaporutu River Mouth Part 2.slpk). Scene layer package (.slpk) format was chosen to optimize the large file size of these 3D models. The corresponding ASCII files (.xyz format) of the computer models have the coordinates (horizontal) and elevation (vertical) information originating from the Digital Surface Model (DSM). The application of these ASCII files is extremely flexible and can be imported into many standard spatial-based software. The resolution of the 3D models and the DSM are based on the density of the point cloud during their generation in Pix4Dmapper software, which ranged from 20 to 50 cm.

The companion ArcGIS Pro project package (.ppkx format) contains all four 3D computer models. Scientists and students, primarily geologists, can import individual models (.slpk) into their own ArcGIS Pro project, or can directly use the companion ArcGIS Pro project. The summary of the dataset is presented in Table 2. The drone models use the WGS 84 UTM 60S coordinate reference system. When opening the ArcGIS project, users must have sufficient high-end computer capacity to display the drone models and import the ASCII files for Digital Surface Model generation.

**Table 2:** Description of the dataset.

Item	Description
3D computer models – scene layer packages	
1. Site 1 Battleship Promontory Close up.slpk	3D computer model of Site 1 – close up view
2. Site 1 Battleship Promontory Overview.slpk	3D model of Site 1 - overview
3.Site 2 Ferry Sandstone.slpk	3D model of Site 2
4.Site 3 Jam Roll part 1.slpk	3D model of Site 3 - first part
5. Site 3 Jam Roll part 2.slpk	3D model of Site 3 - second part
6. Site 4 Tongaporutu River Mouth part 1.slpk	3D model of Site 4 - first part
7. Site 4 Tongaporutu River Mouth part 2.slpk	3D model of Site 4 - second part
Digital Surface Model (DSM) – ASCII xyz	
1. Site 1 Battleship Promontory Close up_i.xyz	DSM for Site 1 - close up view part 1
2. Site 1 Battleship Promontory Close up_ii.xyz	DSM for Site 1 - close up view part 2
3. Site 1 Battleship Promontory Overview.xyz	DSM for Site 1 - overview
4. Site 2 Ferry Sandstone.xyz	DSM for Site 2
5. Site 3 Jam Roll part 1_i.xyz	DSM for Site 3 - part 1
6. Site 3 Jam Roll part 1_ii.xyz	DSM for Site 3 - part 1 continue
7. Site 3 Jam Roll part 2.xyz	DSM for Site 3 - part 2
8. Site 4 Tongaporutu River Mouth part 1_i.xyz	DSM for Site 4 – part 1
9. Site 4 Tongaporutu River Mouth part 1_ii.xyz	DSM for Site 4 - part 1 continue
10.Site 4 Tongaporutu River Mouth part 2.xyz	DSM for Site 4 - part 2
ArcGIS Pro (version 3.0.3) project packages	
1. Sites 1 and 2 3D computer models	ArcGIS Pro project of the 3D computer model for Sites 1 and 2
2. Sites 3 and 4 3D computer models	ArcGIS Pro project of the 3D computer model for Sites 3 and 4

The 3D computer models generated herein are extremely valuable as they enhance the analysis potential of large-scale geological layers, which offers more precise and detailed representations of strata compared to traditionalphotographs and field observations. This will aid geologists, geophysicists, and engineers in gaining a better understanding of the stratigraphic and structural characteristics of formations deposited by ancient deep-water sedimentary systems. Furthermore, the data's utility extends to facilitating virtual fieldwork and field trips by researchers and students, which is advantageous for analyzing areas that are challenging to access due to high elevation, rugged terrain, or locations prone to periodic submersion during high tides. Particularly, these 3D models become invaluable in situations where travel restrictions, such as due to COVID-19, or physical constraints prevent traditional fieldwork from being undertaken.

In addition to the dataset's value for analysis and access, it is also especially important for the digital preservation of geological data and observations. The 3D computer models serve as enduring digital records of these globally significant geological outcrops, which is crucial for the Mount Messenger Formation along the west coast of the North Island of New Zealand, given their vulnerability to coastal erosion and substantial vegetation cover.

Beyond the primary users, this dataset also proves beneficial for other scientists and students engaged in sedimentology, stratigraphy, and sediment basin analysis of paleo deep-water sedimentary deposits. Researchers and industry practitioners may also find utility in this dataset for flow modeling of aquifers, petroleum reservoirs, and carbon capture and geostorage intervals. The 3D computer models function as analogs to similar reservoir systems globally, aiding in estimating flow properties such as porosity and permeability distributions.

Finally, this dataset can be used to support coastal geological and geomorphological research, offering insights into the development of coastal erosion over time. For example, outcrops at specific sites along the coast experience constant erosion from marine processes, impacting the landscape between visits. Beyond coastal studies, the dataset is versatile, applying to environmental and geotechnical analyses. The outcrop models contribute to studies of landslides, exemplified by the outcrop at a specific site adjacent to State Highway 3, an area prone to landslides and rock falls with consequential environmental, safety, and road closure implications.

## 4 Experimental Design, Materials and Methods

The acquisition of the dataset was divided into three stages:

1. Planning:

Planning the drone surveys involved a pre-survey reconnaissance trip to the outcrop sites to assess the feasibility of conducting drone survey operations at the proposed locations. The reconnaissance was carried out in April 2023. Once the drone survey operations were deemed feasible, the drone survey routes and specific locations were planned and necessary permissions from the Department of Conservation, the New Zealand Government, and a private landowner were sought. Once permissions were granted, drone operations were scheduled to account for weather, daylight, and tidal conditions at coastal locations (Sites 3 and 4).

2. Drone survey:

Drone surveys were conducted on the 16th and 17th of June, 12th of August, and 28th and 29th of October 2023. Drone images were captured using programmed flight paths operating on the WGS 84 UTM coordinate reference system and EGM 96 Geoid. RTK and Leica GPS systems were linked to the drone for precise geolocation. The GPS accuracy ranged from 10 to 30 cm. The flight paths were planned to front-face the outcrops, and the distance from the outcrops at Sites 1, 3 and 4 was kept in the range of approximately 3–7 m. However, the drone distance from the outcrop at Site 2 (Fig. 2B) was kept at approximately 15–20 m due to proximity to the road. Due to access permissions, at Site 3, the drone was not allowed to fly over the outcrops, except along the beach section (Fig. 2C). The focal length of the drone camera is 24 mm and images have 20-megapixel resolution. Survey images overlap by 80–90%. Drone survey operations were conducted per the rules and regulations of the Aviation Security Service, New Zealand, and the University of Waikato.

3. 3D outcrop model construction

Approximately three thousand drone images (in jpeg format) were captured during drone surveys. Quality checks of the images ensured that they adequately captured the areas of interest. Minimum preprocessing of images was undertaken, such as colour correction and contrast enhancement. However, 95% of the drone images did not require preprocessing.

**Fig. 3:**
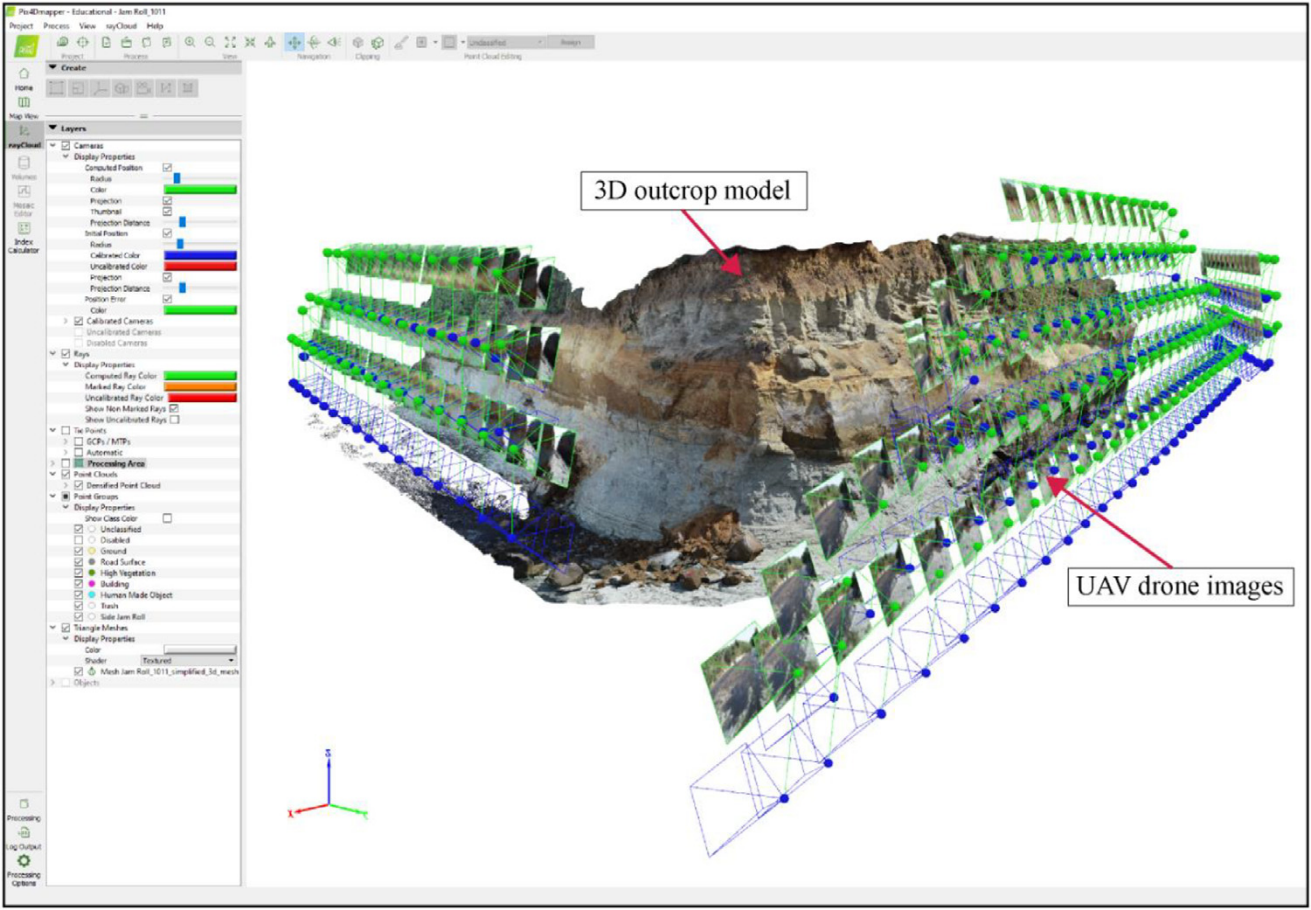
Outcrop 3D computer model generation in Pix4Dmapper. The geolocated UAV drone images overlap by >80% to create a dense point cloud to generate the outcrop model.

**Fig. 4:**
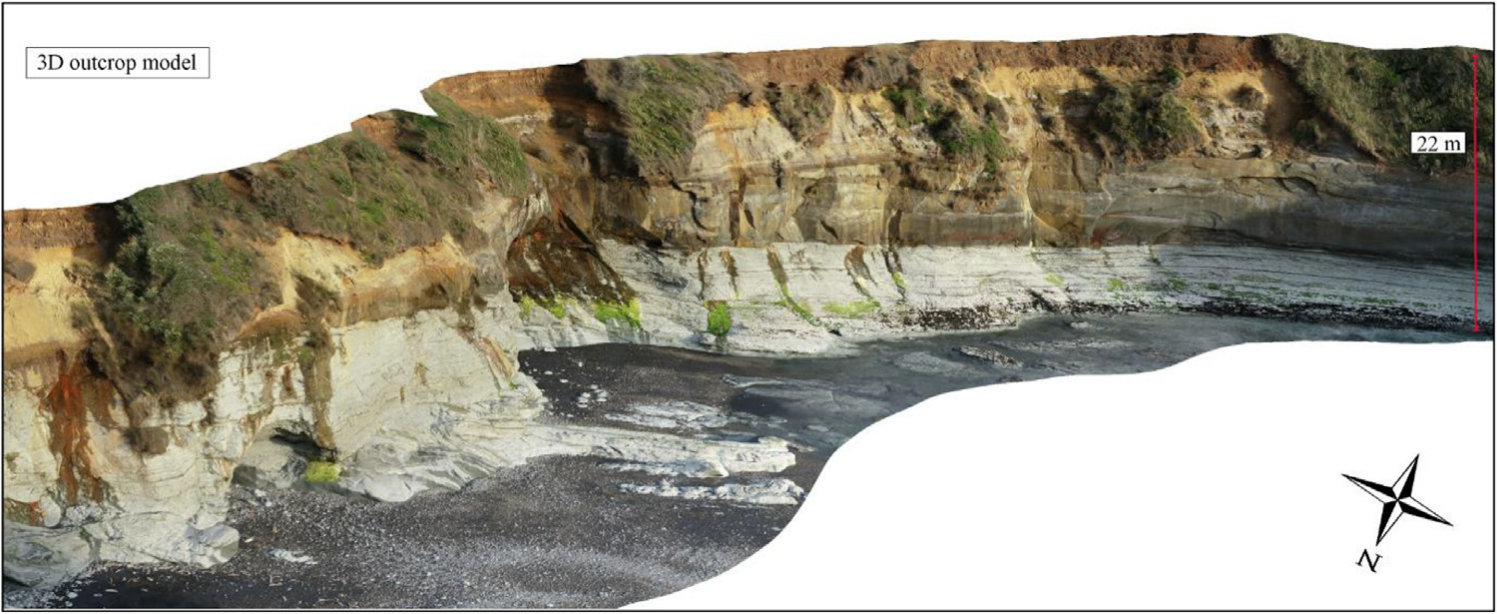
An example of the 3D outcrop model for a part of Site 3: Jam Roll section.

The 3D outcrop drone models were constructed in Pix4Dmapper (version 4.4.12) (Fig. 3). At each outcrop site, the software was used to generate dense 3D point clouds, digital surface models (DSMs), digital terrain models (DTMs), triangle meshes, and orthomosaic images of the outcrops (i.e., 3D models). Constructing individual 3D models took roughly 3–5 h to complete, and the processing reports are attached in the Supplementary Data. Once the 3D outcrop models were constructed, they were exported out of Pix4Dmapper in ArcGIS Pro (version 3.0.3) Scene Layer Package format (.slpk) and loaded into ArcGIS Pro for further geological analysis. An example of the 3D outcrop model is shown in Fig. 4. Accordingly, the corresponding Digital Surface Models are exported to ASCII (.xyz format).

## Limitations

Limitations of 3D computer models built using UAV drone imagery are primarily a function of drone image resolution and their associated models (which in this case ranges from 20 to 50 cm), based on the density of point clouds at certain locations within the models. The GPS accuracy recorded in the field ranged from 10 to 30 cm.

Weather conditions were another limiting factor, especially in terms of adequate sun exposure to capture clear imagery. For example, small parts of the outcrop were not illuminated by the sun to a satisfactory level. However, this did not hinder our objective to conduct geological analysis because the sandstone beds are relatively easy to identify, even under low lighting. Other limitations for drone images resulted from them being captured on different days and times of day, leaving sun-exposure intensity sometimes inconsistent.

## Ethics Statement

We have read and followed the ethical requirements for publication in Data in Brief, and this work did not involve human subjects, animal experiments, or any data collected from social media platforms.

## CRediT Author Statement

Erman Kamaruzaman, Andrew La Croix and Peter Kamp: Conceptualisation, Methodology, Writing and Editing. Erman Kamaruzaman: Software, Data Investigation and Visualisation. Erman Kamaruzaman and Andrew La Croix: Data acquisition.

## Data Availability

3D drone outcrop models of the Mount Messenger Formation, New Zealand (Original data) (Dataverse)

## Appendix Supplementary materials

Image, application 1
