# Supplementary material for: Dataset of 3D computer models of Late Miocene Mount Messenger Formation outcrops in New Zealand, built with UAV drones
Source: Data Brief. 2024 Jan 9;52:110035. doi: 10.1016/j.dib.2024.110035 (PMC10827393; doi:10.1016/j.dib.2024.110035)
Supplement: Supplementary file 1 [file mmc1.pdf]

Quality Report

Generated with Pix4Dmapper version 4.4.12

Important: Click on the different icons for:

Help to analyze the results in the Quality Report

Additional information about the sections

Click here for additional tips to analyze the Quality Report

Summary

Project	Battleship_1511
Processed	2023-11-15 20:2[illegible]
Camera Model Name(s)	FC6310R_8.8_5472x3648 (RGB), FC6310R_8.8_4864x3648 (RGB)
Average Ground Sampling Distance (GSD)	1.11 cm / 0.44 in
Area Covered	0.056 km^2 / 5.5764 ha / 0.02 sq. mi. / 13.7868 acres
Time for Initial Processing (without report)	57m:20s

Quality Check

**Images**	median of 61034 keypoints per image	
**Dataset**	420 out of 420 images calibrated (100%), all images enabled, 2 blocks	
**Camera Optimization**	0.76% relative difference between initial and optimized internal camera parameters	
**Matching**	median of 27406 matches per calibrated image	
**Georeferencing**	yes, no 3D GCP	

Preview

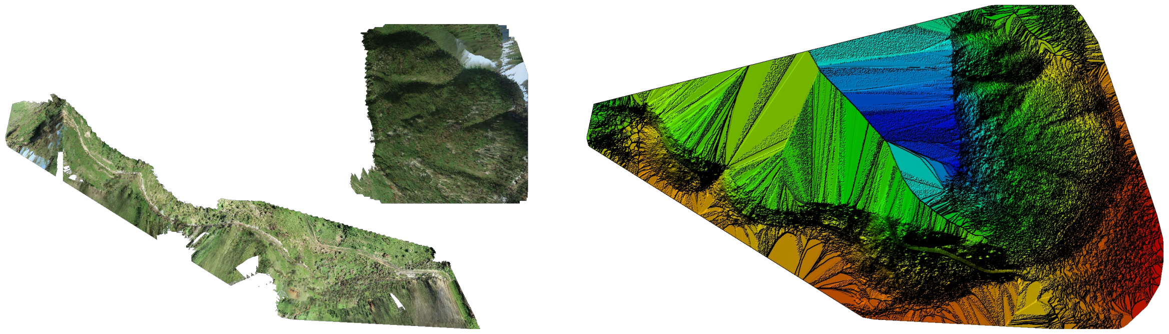

Figure 1: Orthomosaic and the corresponding sparse Digital Surface Model (DSM) before densification.

Calibration Details

Number of Calibrated Images	420 out of 420
Number of Geolocated Images	420 out of 420

Initial Image Positions

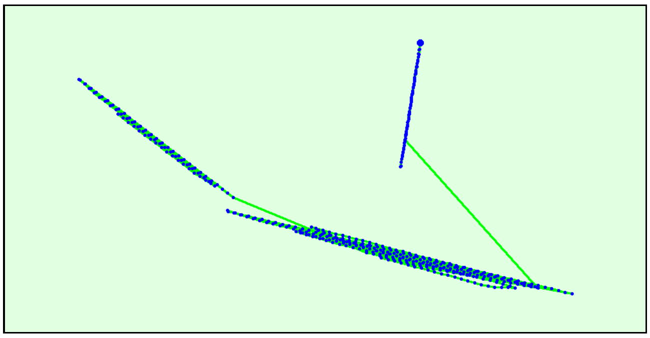

Figure 2: Top view of the initial image position. The green line follows the position of the images in time starting from the large blue dot.

Computed Image/GCPs/Manual Tie Points Positions

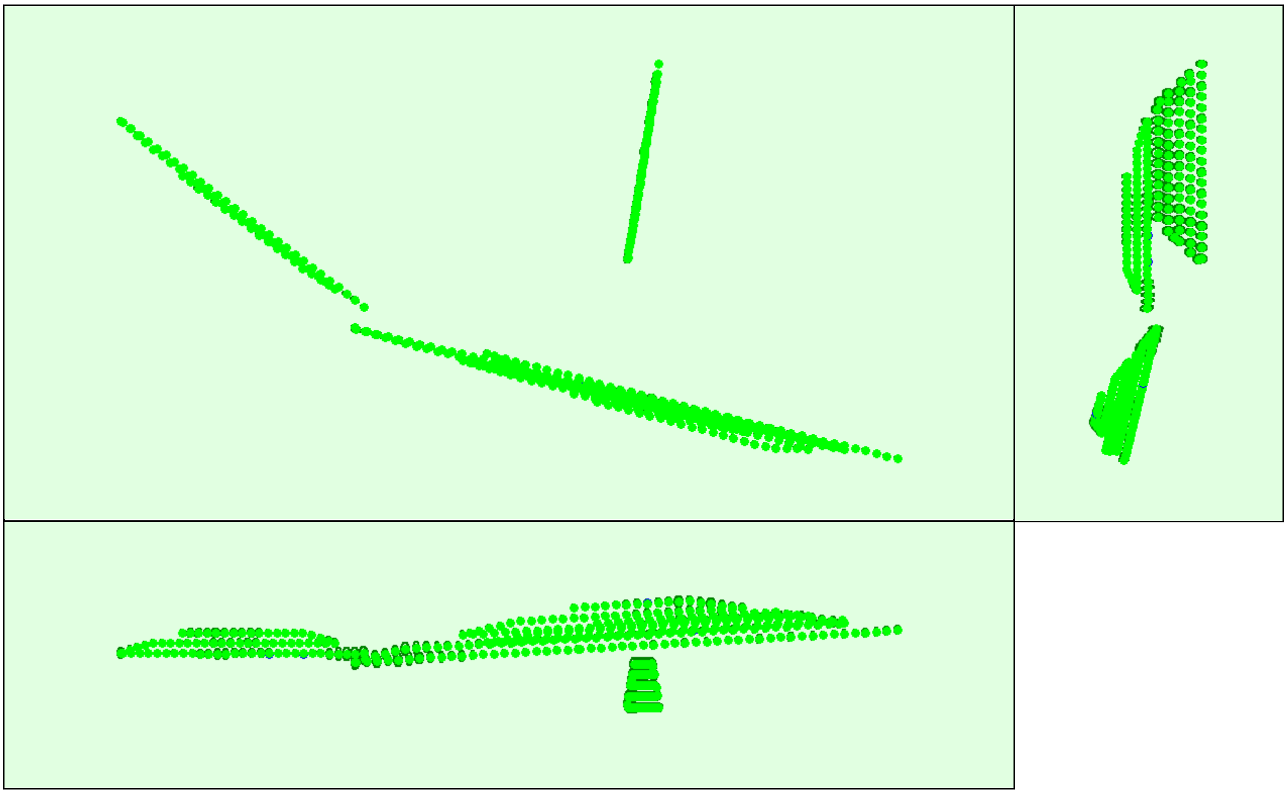

Uncertainty ellipses 1000x magnified

Figure 3: Offset between initial (blue dots) and computed (green dots) image positions as well as the offset between the GCPs initial positions (blue crosses) and their computed positions (green crosses) in the top-view (XY plane), front-view (XZ plane), and side-view (YZ plane). Dark green ellipses indicate the absolute position uncertainty of the bundle block adjustment result.

Absolute camera position and orientation uncertainties

	X[m]	Y[m]	Z[m]	Omega [degree]	Phi [degree]	Kappa [degree]
Mean	0.001	0.001	0.002	0.039	0.003	0.005
Sigma	0.000	0.000	0.000	0.075	0.002	0.009

Overlap

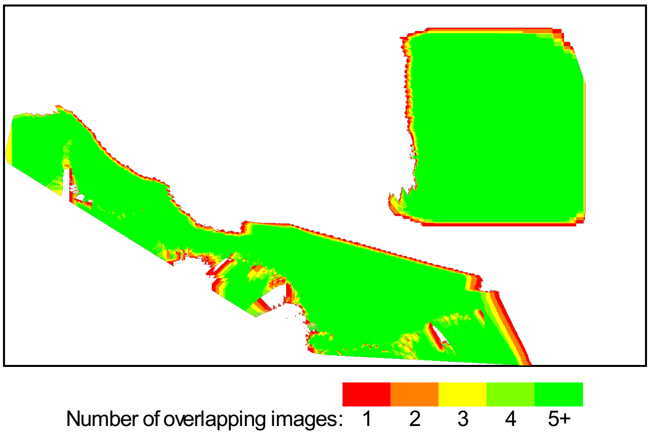

Figure 4: Number of overlapping images computed for each pixel of the orthomosaic.
Red and yellow areas indicate low overlap for which poor results may be generated. Green areas indicate an overlap of over 5 images for every pixel. Good quality results will be generated as long as the number of keypoint matches is also sufficient for these areas (see Figure 5 for keypoint matches).

Bundle Block Adjustment Details

Number of 2D Keypoint Observations for Bundle Block Adjustment	11442831
Number of 3D Points for Bundle Block Adjustment	2855826
Mean Reprojection Error [pixels]	0.149

Internal Camera Parameters

FC6310R_8.8_5472x3648 (RGB). Sensor Dimensions: 12.833 [mm] x 8.556 [mm]

EXIF ID: FC6310R_8.8_5472x3648

	Focal Length	Principal Point x	Principal Point y	R1	R2	R3	T1	T2
Initial Values	3658.300 [pixel]<br>8.580 [mm]	2722.500 [pixel]<br>6.385 [mm]	1835.100 [pixel]<br>4.304 [mm]	-0.269	0.112	-0.033	0.000	-0.001
Optimized Values	3635.056 [pixel]<br>8.525 [mm]	2729.728 [pixel]<br>6.402 [mm]	1843.503 [pixel]<br>4.324 [mm]	-0.267	0.108	-0.030	-0.000	-0.000
Uncertainties (Sigma)	1.031 [pixel]<br>0.002 [mm]	0.746 [pixel]<br>0.002 [mm]	0.636 [pixel]<br>0.001 [mm]	0.000	0.000	0.000	0.000	0.000

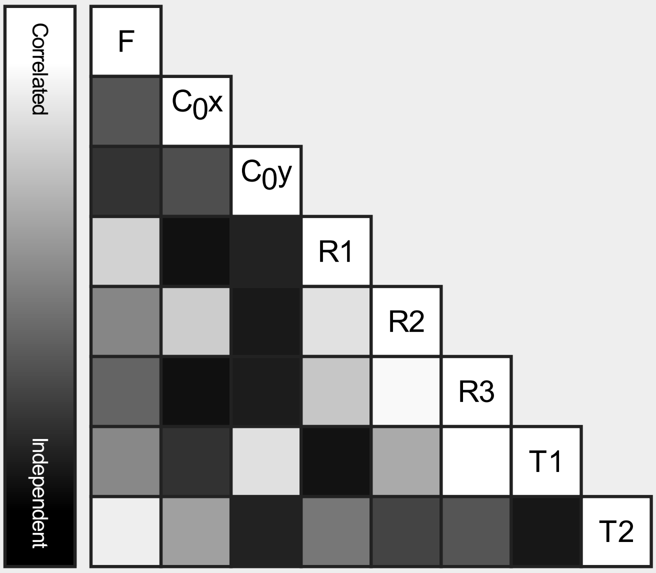

The correlation between camera internal parameters determined by the bundle adjustment. White indicates a full correlation between the parameters, ie. any change in one can be fully compensated by the other. Black indicates that the parameter is completely independent, and is not affected by other parameters.

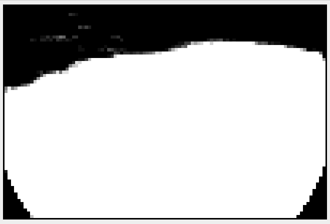

The number of Automatic Tie Points (ATPs) per pixel, averaged over all images of the camera model, is color coded between black and white. White indicates that, on average, more than 16 ATPs have been extracted at the pixel location. Black indicates that, on average, 0 ATPs have been extracted at the pixel location. Click on the image to the see the average direction and magnitude of the re-projection error for each pixel. Note that the vectors are scaled for better visualization. The scale bar indicates the magnitude of 1 pixel error.

Internal Camera Parameters

FC6310R_8.8_4864x3648 (RGB). Sensor Dimensions: 11.407 [mm] x 8.556 [mm]

EXIF ID: FC6310R_8.8_4864x3648

	Focal Length	Principal Point x	Principal Point y	R1	R2	R3	T1	T2
Initial Values	3666.840 [pixel] 8.600 [mm]	2420.300 [pixel] 5.676 [mm]	1835.990 [pixel] 4.306 [mm]	-0.270	0.112	-0.032	0.000	-0.001
Optimized Values	3634.262 [pixel] 8.523 [mm]	2421.510 [pixel] 5.679 [mm]	1836.980 [pixel] 4.308 [mm]	-0.268	0.113	-0.034	-0.000	-0.001
Uncertainties (Sigma)	0.086 [pixel] 0.000 [mm]	0.111 [pixel] 0.000 [mm]	0.064 [pixel] 0.000 [mm]	0.000	0.000	0.000	0.000	0.000

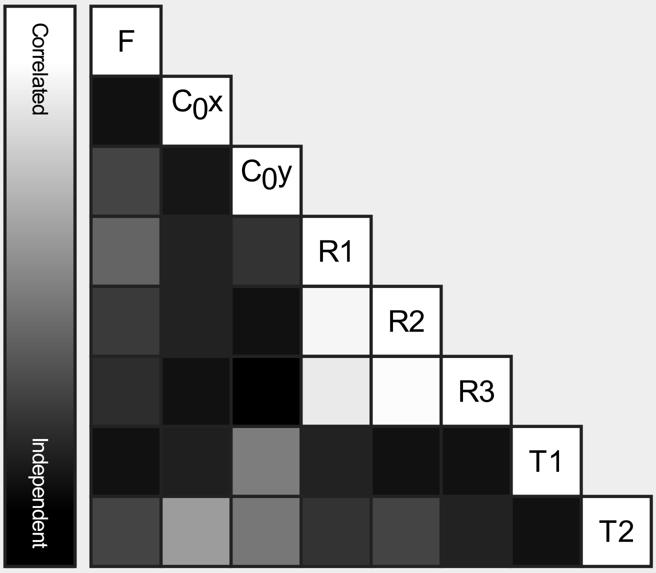

The correlation between camera internal parameters determined by the bundle adjustment. White indicates a full correlation between the parameters, ie. any change in one can be fully compensated by the other. Black indicates that the parameter is completely independent, and is not affected by other parameters.

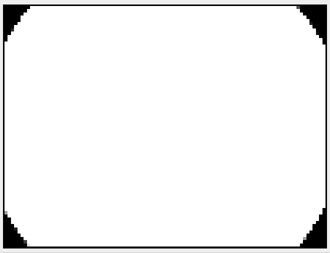

The number of Automatic Tie Points (ATPs) per pixel, averaged over all images of the camera model, is color coded between black and white. White indicates that, on average, more than 16 ATPs have been extracted at the pixel location. Black indicates that, on average, 0 ATPs have been extracted at the pixel location. Click on the image to the see the average direction and magnitude of the re-projection error for each pixel. Note that the vectors are scaled for better visualization. The scale bar indicates the magnitude of 1 pixel error.

2D Keypoints Table

	Number of 2D Keypoints per Image	Number of Matched 2D Keypoints per Image
Median	61034	27406
Min	37061	12087
Max	77059	46135
Mean	57882	27245

2D Keypoints Table for Camera FC6310R_8.8_5472x3648 (RGB)

	Number of 2D Keypoints per Image	Number of Matched 2D Keypoints per Image
Median	45092	29919

Min	37061	22999
Max	53814	35043
Mean	44992	30369

2D Keypoints Table for Camera FC6310R_8.8_4864x3648 (RGB)

	Number of 2D Keypoints per Image	Number of Matched 2D Keypoints per Image
Median	61990	26441
Min	39527	12087
Max	77059	46135
Mean	60962	26498

Median / 75% / Maximal Number of Matches Between Camera Models

	FC6310R_8.8_547...(RGB)	FC6310R_8.8_486...(RGB)
FC6310R_8.8_5472x3648 (RGB)	7098 / 9820 / 23554	
FC6310R_8.8_4864x3648 (RGB)		474 / 1899 / 34820

3D Points from 2D Keypoint Matches

	Number of 3D Points Observed
In 2 Images	1594374
In 3 Images	469430
In 4 Images	226465
In 5 Images	132921
In 6 Images	88211
In 7 Images	62682
In 8 Images	46924
In 9 Images	36155
In 10 Images	28531
In 11 Images	23346
In 12 Images	19322
In 13 Images	15861
In 14 Images	13403
In 15 Images	11245
In 16 Images	9598
In 17 Images	8563
In 18 Images	7290
In 19 Images	6342
In 20 Images	5558
In 21 Images	4846
In 22 Images	4314
In 23 Images	3819
In 24 Images	3276
In 25 Images	2868
In 26 Images	2616
In 27 Images	2327
In 28 Images	2056
In 29 Images	1912
In 30 Images	1723
In 31 Images	1522
In 32 Images	1380
In 33 Images	1291
In 34 Images	1187
In 35 Images	1022
In 36 Images	898
In 37 Images	808
In 38 Images	749
In 39 Images	727

In 40 Images	630
In 41 Images	601
In 42 Images	524
In 43 Images	533
In 44 Images	486
In 45 Images	454
In 46 Images	458
In 47 Images	422
In 48 Images	351
In 49 Images	358
In 50 Images	325
In 51 Images	309
In 52 Images	270
In 53 Images	287
In 54 Images	267
In 55 Images	261
In 56 Images	272
In 57 Images	237
In 58 Images	207
In 59 Images	222
In 60 Images	185
In 61 Images	170
In 62 Images	196
In 63 Images	171
In 64 Images	157
In 65 Images	160
In 66 Images	138
In 67 Images	146
In 68 Images	110
In 69 Images	118
In 70 Images	127
In 71 Images	113
In 72 Images	133
In 73 Images	94
In 74 Images	97
In 75 Images	91
In 76 Images	97
In 77 Images	75
In 78 Images	79
In 79 Images	75
In 80 Images	97
In 81 Images	161

2D Keypoint Matches

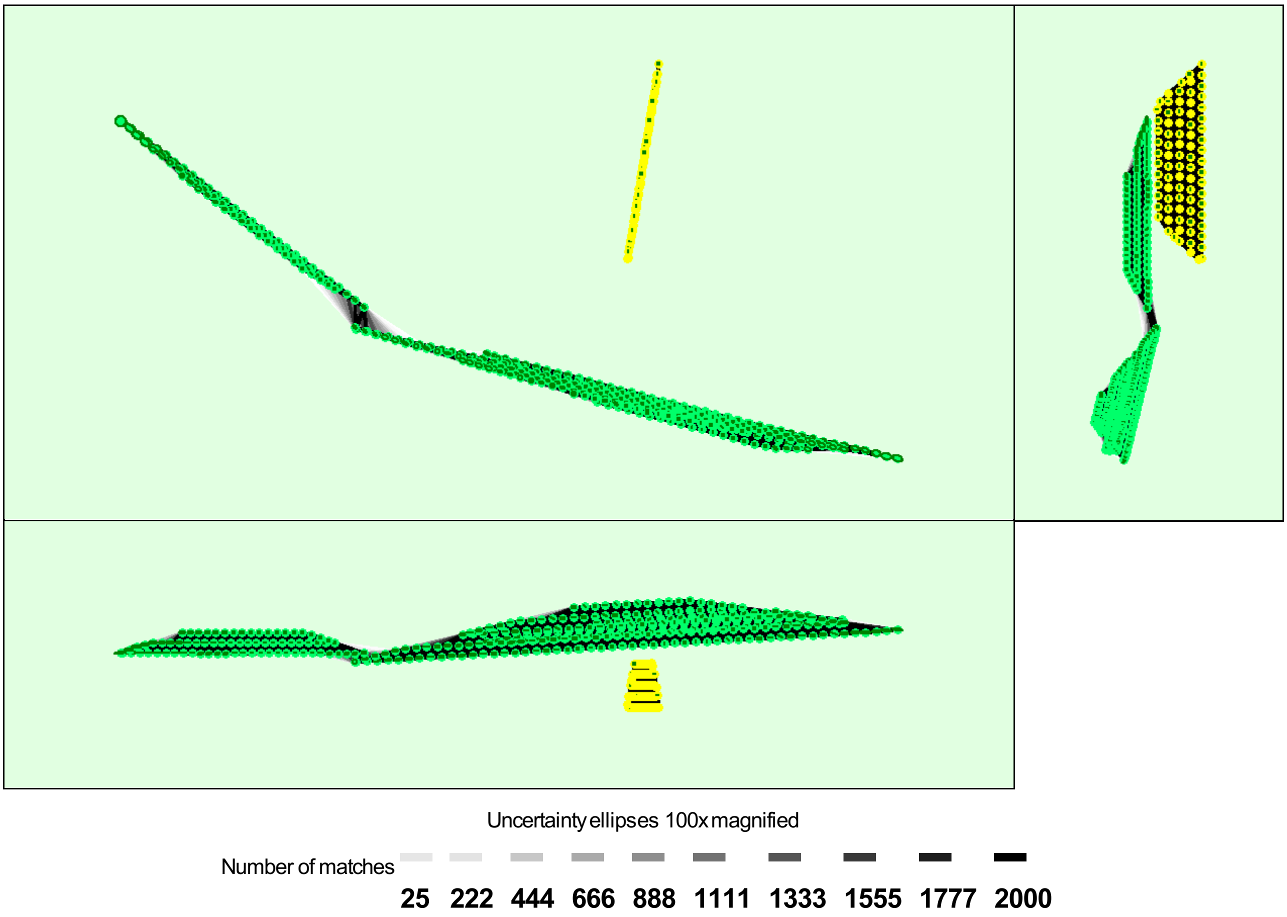

Figure 5: Computed image positions with links between matched images. The darkness of the links indicates the number of matched 2D keypoints between the images. Bright links indicate weak links and require manual tie points or more images. Dark green ellipses indicate the relative camera position uncertainty of the bundle block adjustment result.

Relative camera position and orientation uncertainties

	X[m]	Y[m]	Z[m]	Omega [degree]	Phi [degree]	Kappa [degree]
Mean	0.007	0.006	0.003	0.037	0.007	0.006
Sigma	0.005	0.004	0.001	0.031	0.002	0.004

Geolocation Details

Absolute Geolocation Variance

Min Error [m]	Max Error [m]	Geolocation Error X [%]	Geolocation Error Y [%]	Geolocation Error Z [%]
-	-0.04	0.00	0.00	0.00
-0.04	-0.03	0.00	0.00	0.00
-0.03	-0.02	0.00	0.00	0.00
-0.02	-0.01	0.24	0.00	1.19
-0.01	-0.01	1.67	1.90	11.43
-0.01	0.00	49.52	48.57	35.24
0.00	0.01	45.95	47.62	40.71
0.01	0.01	2.62	1.90	10.24
0.01	0.02	0.00	0.00	0.95
0.02	0.03	0.00	0.00	0.00
0.03	0.04	0.00	0.00	0.24
0.04	-	0.00	0.00	0.00
**Mean [m]**		-0.000020	-0.000053	0.000072

**Sigma [m]**	0.003575	0.003575	0.006504
**RMS Error [m]**	0.003576	0.003575	0.006504

Min Error and Max Error represent geolocation error intervals between -1.5 and 1.5 times the maximum accuracy of all the images. Columns X, Y, Z show the percentage of images with geolocation errors within the predefined error intervals. The geolocation error is the difference between the initial and computed image positions. Note that the image geolocation errors do not correspond to the accuracy of the observed 3D points.

Relative Geolocation Variance

Relative Geolocation Error	Images X [%]	Images Y [%]	Images Z [%]
[-1.00, 1.00]	99.29	99.29	99.76
[-2.00, 2.00]	100.00	100.00	100.00
[-3.00, 3.00]	100.00	100.00	100.00
**Mean of Geolocation Accuracy [m]**	0.010337	0.010337	0.020955
**Sigma of Geolocation Accuracy [m]**	0.000687	0.000687	0.000696

Images X, Y, Z represent the percentage of images with a relative geolocation error in X, Y, Z.

Geolocation Orientational Variance	RMS [degree]
Omega	3.163
Phi	4.982
Kappa	5.482

Geolocation RMS error of the orientation angles given by the difference between the initial and computed image orientation angles.

Initial Processing Details

System Information

Hardware	CPU: Intel(R) Core(TM) i7-10700 CPU @ 2.90GHz<br>RAM: 64GB<br>GPU: Microsoft Remote Display Adapter (Driver: unknown), Microsoft Remote Display Adapter (Driver: unknown), Microsoft Remote Display Adapter (Driver: unknown), Microsoft Remote Display Adapter (Driver: unknown), Microsoft Remote Display Adapter (Driver: unknown), Microsoft Remote Display Adapter (Driver: unknown), Microsoft Remote Display Adapter (Driver: unknown), Microsoft Remote Display Adapter (Driver: unknown), Microsoft Remote Display Adapter (Driver: unknown), Microsoft Remote Display Adapter (Driver: unknown), Microsoft Remote Display Adapter (Driver: unknown), Microsoft Remote Display Adapter (Driver: unknown), Microsoft Remote Display Adapter (Driver: unknown), Microsoft Remote Display Adapter (Driver: unknown), Microsoft Remote Display Adapter (Driver: unknown), Microsoft Remote Display Adapter (Driver: unknown)
Operating System	Windows 10 Enterprise, 64-bit

Coordinate Systems

Image Coordinate System	WGS 84
Output Coordinate System	WGS 84 / UTM zone 60S (+25m)

Processing Options

Detected Template	No Template Available
Keypoints Image Scale	Full, Image Scale: 1
Advanced: Matching Image Pairs	Aerial Grid or Corridor
Advanced: Matching Strategy	Use Geometrically Verified Matching: yes
Advanced: Keypoint Extraction	Targeted Number of Keypoints: Automatic
Advanced: Calibration	Calibration Method: Geolocation Based<br>Internal Parameters Optimization: All<br>External Parameters Optimization: All<br>Rematch: Auto, yes

Point Cloud Densification details

Processing Options

Image Scale	multiscale, 1/2 (Half image size, Default)
Point Density	Optimal
Minimum Number of Matches	3
3D Textured Mesh Generation	yes
3D Textured Mesh Settings:	Resolution: High Resolution<br>Color Balancing: no
LOD	Generated: no
Advanced: 3D Textured Mesh Settings	Sample Density Divider: 1
Advanced: Image Groups	group1
Advanced: Use Processing Area	yes
Advanced: Use Annotations	yes
Time for Point Cloud Densification	01h:15m:18s
Time for Point Cloud Classification	NA
Time for 3D Textured Mesh Generation	30m:34s

Results

Number of Generated Tiles	3
Number of 3D Densified Points	34313590
Average Density (per m^3)	1364.83

DSM, Orthomosaic and Index Details

Processing Options

DSM and Orthomosaic Resolution	1 x GSD (1.11 [cm/pixel])
DSM Filters	Noise Filtering: yes<br>Surface Smoothing: yes, Type: Sharp
Raster DSM	Generated: yes<br>Method: Inverse Distance Weighting<br>Merge Tiles: yes
Orthomosaic	Generated: yes<br>Merge Tiles: yes<br>GeoTIFF Without Transparency: no<br>Google Maps Tiles and KML: no
Time for DSM Generation	29m:14s
Time for Orthomosaic Generation	58m:15s
Time for DTM Generation	00s
Time for Contour Lines Generation	00s
Time for Reflectance Map Generation	00s
Time for Index Map Generation	00s

Quality Report

Generated with Pix4Dmapper version 4.4.12

Important: Click on the different icons for:

Help to analyze the results in the Quality Report

Additional information about the sections

Click here for additional tips to analyze the Quality Report

Summary

Project	Ferry Sst_1506
Processed	2023-06-15 17:53:40
Camera Model Name(s)	FC6310R_8.8_5472x3648 (RGB)
Average Ground Sampling Distance (GSD)	3.32 cm / 1.31 in
Time for Initial Processing (without report)	03h:35m:17s

Quality Check

**Images**	median of 50620 keypoints per image	
**Dataset**	425 out of 425 images calibrated (100%), all images enabled	
**Camera Optimization**	0.7% relative difference between initial and optimized internal camera parameters	
**Matching**	median of 24043.9 matches per calibrated image	
**Georeferencing**	yes, no 3D GCP	

Calibration Details

Number of Calibrated Images	425 out of 425
Number of Geolocated Images	425 out of 425

Initial Image Positions

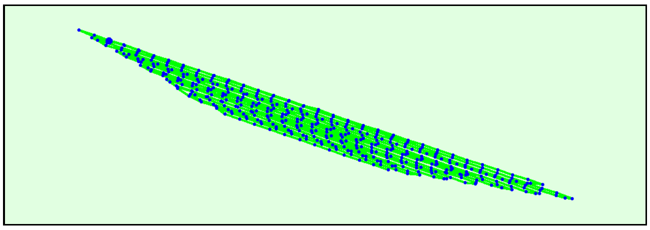

Figure 2: Top view of the initial image position. The green line follows the position of the images in time starting from the large blue dot.

Computed Image/GCPs/Manual Tie Points Positions

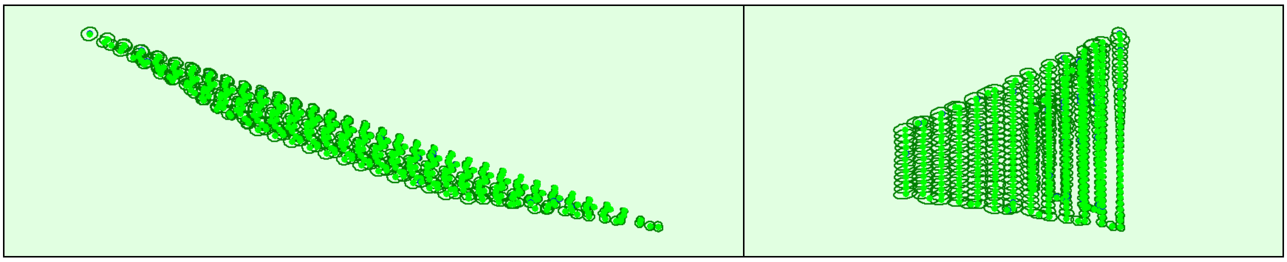

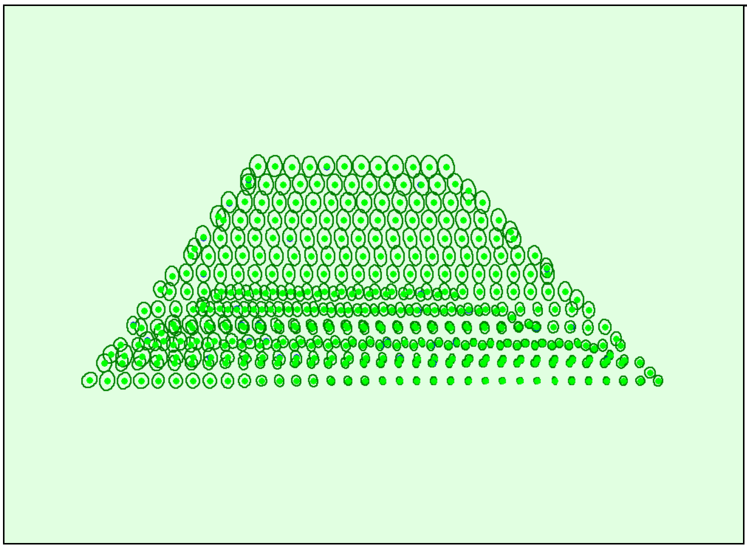

Uncertainty ellipses 1000x magnified

Figure 3: Offset between initial (blue dots) and computed (green dots) image positions as well as the offset between the GCPs initial positions (blue crosses) and their computed positions (green crosses) in the top-view (XY plane), front-view (XZ plane), and side-view (YZ plane). Dark green ellipses indicate the absolute position uncertainty of the bundle block adjustment result.

Absolute camera position and orientation uncertainties

	X [m]	Y [m]	Z [m]	Omega [degree]	Phi [degree]	Kappa [degree]
Mean	0.002	0.001	0.002	0.002	0.002	0.001
Sigma	0.000	0.000	0.001	0.000	0.000	0.000

Bundle Block Adjustment Details

Number of 2D Keypoint Observations for Bundle Block Adjustment	9703722
Number of 3D Points for Bundle Block Adjustment	1970105
Mean Reprojection Error [pixels]	0.184

Internal Camera Parameters

FC6310R_8.8_5472x3648 (RGB). Sensor Dimensions: 12.833 [mm] x 8.556 [mm]

EXIF ID: FC6310R_8.8_5472x3648

	Focal Length	Principal Point x	Principal Point y	R1	R2	R3	T1	T2
Initial Values	3658.300 [pixel] 8.580 [mm]	2722.500 [pixel] 6.385 [mm]	1835.100 [pixel] 4.304 [mm]	-0.269	0.112	-0.033	0.000	-0.001
Optimized Values	3632.576 [pixel] 8.519 [mm]	2723.891 [pixel] 6.388 [mm]	1844.755 [pixel] 4.326 [mm]	-0.267	0.109	-0.031	-0.000	-0.001
Uncertainties (Sigma)	0.068 [pixel] 0.000 [mm]	0.183 [pixel] 0.000 [mm]	0.067 [pixel] 0.000 [mm]	0.000	0.000	0.000	0.000	0.000

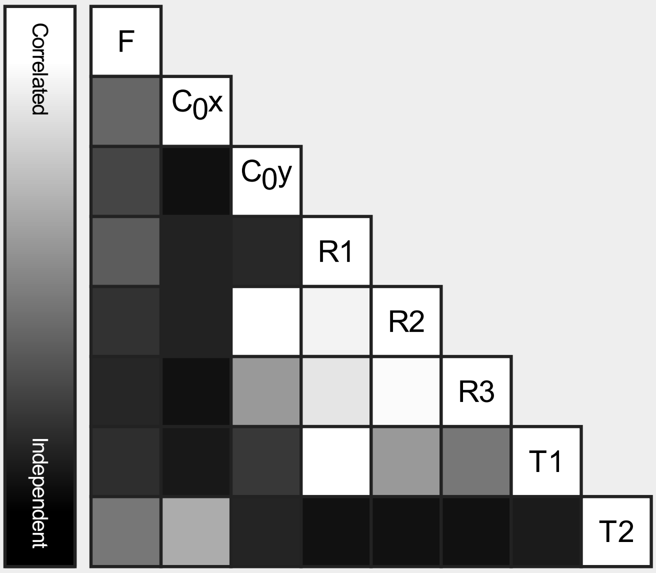

The correlation between camera internal parameters determined by the bundle adjustment. White indicates a full correlation between the parameters, ie. any change in one can be fully compensated by the other. Black indicates that the parameter is completely independent, and is not affected by other parameters.

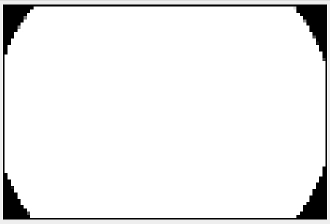

The number of Automatic Tie Points (ATPs) per pixel, averaged over all images of the camera model, is color coded between black and white. White indicates that, on average, more than 16 ATPs have been extracted at the pixel location. Black indicates that, on average, 0 ATPs have been extracted at the pixel location. Click on the image to the see the average direction and magnitude of the re-projection error for each pixel. Note that the vectors are scaled for better visualization. The scale bar indicates the magnitude of 1 pixel error.

2D Keypoints Table

	Number of 2D Keypoints per Image	Number of Matched 2D Keypoints per Image
Median	50620	24044
Min	26176	10065
Max	73483	37227
Mean	50992	22832

3D Points from 2D Keypoint Matches

	Number of 3D Points Observed
In 2 Images	1008670
In 3 Images	340475
In 4 Images	171568
In 5 Images	102120
In 6 Images	67700
In 7 Images	47596
In 8 Images	35533
In 9 Images	26933
In 10 Images	21562
In 11 Images	17278
In 12 Images	14078
In 13 Images	11966
In 14 Images	10010
In 15 Images	8286
In 16 Images	7272
In 17 Images	6351
In 18 Images	5501
In 19 Images	4856
In 20 Images	4326
In 21 Images	3932
In 22 Images	3503
In 23 Images	3192

In 24 Images	2889
In 25 Images	2564
In 26 Images	2271
In 27 Images	2111
In 28 Images	2014
In 29 Images	1837
In 30 Images	1674
In 31 Images	1541
In 32 Images	1385
In 33 Images	1335
In 34 Images	1232
In 35 Images	1182
In 36 Images	1072
In 37 Images	965
In 38 Images	923
In 39 Images	839
In 40 Images	760
In 41 Images	746
In 42 Images	749
In 43 Images	744
In 44 Images	642
In 45 Images	606
In 46 Images	602
In 47 Images	539
In 48 Images	500
In 49 Images	521
In 50 Images	482
In 51 Images	461
In 52 Images	384
In 53 Images	445
In 54 Images	350
In 55 Images	396
In 56 Images	363
In 57 Images	365
In 58 Images	338
In 59 Images	287
In 60 Images	306
In 61 Images	280
In 62 Images	286
In 63 Images	260
In 64 Images	264
In 65 Images	241
In 66 Images	243
In 67 Images	228
In 68 Images	242
In 69 Images	207
In 70 Images	228
In 71 Images	229
In 72 Images	190
In 73 Images	180
In 74 Images	201
In 75 Images	199
In 76 Images	186
In 77 Images	149
In 78 Images	172
In 79 Images	170
In 80 Images	161
In 81 Images	153
In 82 Images	127

In 83 Images	130
In 84 Images	155
In 85 Images	161
In 86 Images	110
In 87 Images	127
In 88 Images	113
In 89 Images	109
In 90 Images	126
In 91 Images	119
In 92 Images	109
In 93 Images	115
In 94 Images	94
In 95 Images	100
In 96 Images	90
In 97 Images	93
In 98 Images	88
In 99 Images	92
In 100 Images	75
In 101 Images	81
In 102 Images	81
In 103 Images	78
In 104 Images	64
In 105 Images	85
In 106 Images	68
In 107 Images	87
In 108 Images	68
In 109 Images	70
In 110 Images	65
In 111 Images	66
In 112 Images	60
In 113 Images	51
In 114 Images	71
In 115 Images	64
In 116 Images	64
In 117 Images	66
In 118 Images	58
In 119 Images	53
In 120 Images	55
In 121 Images	55
In 122 Images	54
In 123 Images	56
In 124 Images	41
In 125 Images	64
In 126 Images	54
In 127 Images	36
In 128 Images	60
In 129 Images	37
In 130 Images	47
In 131 Images	34
In 132 Images	39
In 133 Images	36
In 134 Images	34
In 135 Images	29
In 136 Images	34
In 137 Images	42
In 138 Images	39
In 139 Images	33
In 140 Images	36
In 141 Images	38

In 142 Images	32
In 143 Images	33
In 144 Images	21
In 145 Images	32
In 146 Images	32
In 147 Images	25
In 148 Images	31
In 149 Images	32
In 150 Images	22
In 151 Images	26
In 152 Images	26
In 153 Images	26
In 154 Images	28
In 155 Images	24
In 156 Images	27
In 157 Images	25
In 158 Images	25
In 159 Images	34
In 160 Images	23
In 161 Images	27
In 162 Images	23
In 163 Images	27
In 164 Images	38
In 165 Images	27
In 166 Images	17
In 167 Images	24
In 168 Images	29
In 169 Images	24
In 170 Images	21
In 171 Images	23
In 172 Images	25
In 173 Images	17
In 174 Images	19
In 175 Images	15
In 176 Images	21
In 177 Images	16
In 178 Images	24
In 179 Images	23
In 180 Images	14
In 181 Images	16
In 182 Images	18
In 183 Images	15
In 184 Images	20
In 185 Images	14
In 186 Images	15
In 187 Images	21
In 188 Images	7
In 189 Images	16
In 190 Images	18
In 191 Images	14
In 192 Images	18
In 193 Images	14
In 194 Images	14
In 195 Images	13
In 196 Images	12
In 197 Images	19
In 198 Images	14
In 199 Images	13
In 200 Images	24

In 201 Images	7
In 202 Images	13
In 203 Images	10
In 204 Images	13
In 205 Images	12
In 206 Images	8
In 207 Images	16
In 208 Images	17
In 209 Images	15
In 210 Images	11
In 211 Images	12
In 212 Images	11
In 213 Images	13
In 214 Images	15
In 215 Images	15
In 216 Images	13
In 217 Images	12
In 218 Images	8
In 219 Images	7
In 220 Images	15
In 221 Images	11
In 222 Images	12
In 223 Images	11
In 224 Images	9
In 225 Images	8
In 226 Images	5
In 227 Images	12
In 228 Images	14
In 229 Images	10
In 230 Images	13
In 231 Images	11
In 232 Images	5
In 233 Images	8
In 234 Images	10
In 235 Images	11
In 236 Images	11
In 237 Images	11
In 238 Images	6
In 239 Images	10
In 240 Images	14
In 241 Images	5
In 242 Images	9
In 243 Images	9
In 244 Images	3
In 245 Images	4
In 246 Images	7
In 247 Images	8
In 248 Images	3
In 249 Images	6
In 250 Images	6
In 251 Images	8
In 252 Images	4
In 253 Images	8
In 254 Images	4
In 255 Images	8
In 256 Images	10
In 257 Images	4
In 258 Images	10
In 259 Images	8

In 260 Images	4
In 261 Images	7
In 262 Images	3
In 263 Images	4
In 264 Images	8
In 265 Images	8
In 266 Images	6
In 267 Images	7
In 268 Images	5
In 269 Images	5
In 270 Images	9
In 271 Images	5
In 272 Images	2
In 273 Images	7
In 274 Images	3
In 275 Images	8
In 276 Images	4
In 277 Images	7
In 278 Images	5
In 279 Images	3
In 280 Images	7
In 281 Images	2
In 282 Images	6
In 283 Images	4
In 284 Images	5
In 285 Images	1
In 286 Images	5
In 287 Images	1
In 288 Images	6
In 289 Images	3
In 290 Images	3
In 291 Images	4
In 292 Images	3
In 293 Images	3
In 294 Images	5
In 295 Images	2
In 296 Images	2
In 297 Images	3
In 298 Images	1
In 299 Images	2
In 300 Images	2
In 301 Images	4
In 302 Images	4
In 303 Images	4
In 304 Images	4
In 305 Images	3
In 306 Images	1
In 307 Images	2
In 308 Images	2
In 309 Images	1
In 310 Images	2
In 311 Images	2
In 313 Images	1
In 314 Images	1
In 315 Images	2
In 316 Images	2
In 317 Images	2
In 319 Images	1
In 320 Images	1

In 323 Images	2
In 324 Images	1
In 326 Images	2
In 327 Images	2
In 328 Images	1
In 329 Images	1
In 330 Images	1
In 332 Images	2
In 333 Images	1
In 334 Images	1
In 335 Images	1
In 336 Images	1
In 338 Images	1
In 339 Images	1
In 340 Images	1
In 342 Images	1
In 344 Images	1
In 345 Images	2
In 346 Images	1
In 352 Images	1
In 353 Images	2
In 354 Images	1
In 355 Images	1
In 357 Images	1
In 359 Images	1
In 360 Images	1
In 362 Images	1
In 364 Images	1
In 366 Images	1
In 375 Images	1
In 377 Images	1
In 379 Images	1
In 380 Images	1
In 391 Images	1
In 392 Images	1

2D Keypoint Matches

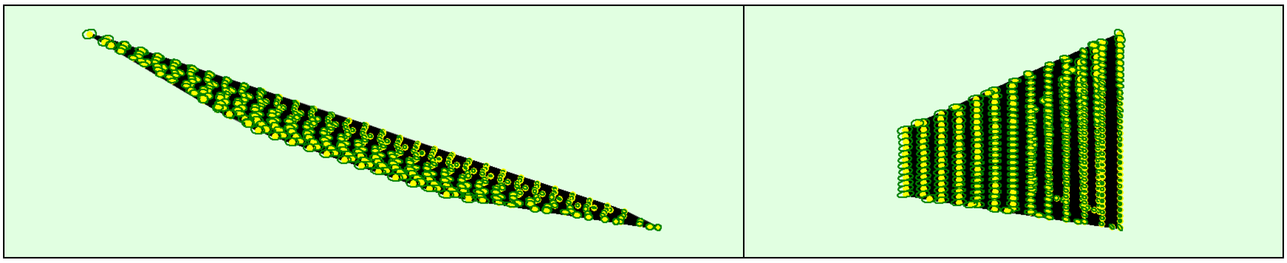

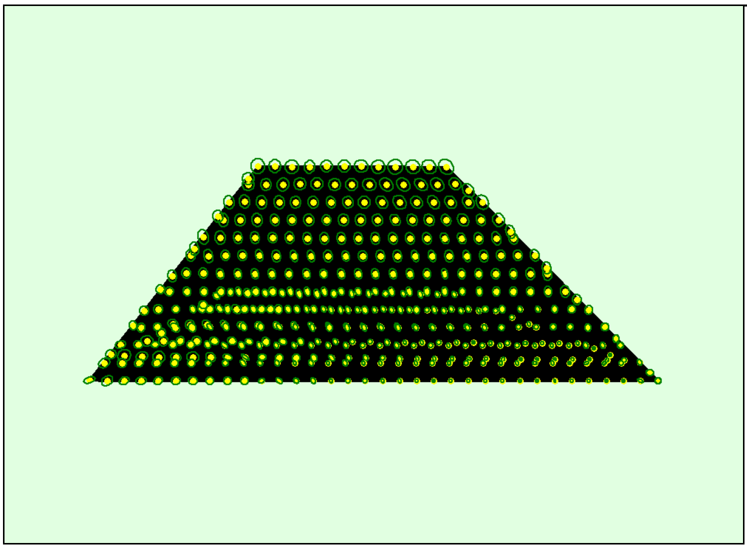

Uncertainty ellipses 500x magnified

Number of matches 25 222 444 666 888 1111 1333 1555 1777 2000

Figure 5: Computed image positions with links between matched images. The darkness of the links indicates the number of matched 2D keypoints between the images. Bright links indicate weak links and require manual tie points or more images. Dark green ellipses indicate the relative camera position uncertainty of the bundle block adjustment result.

Relative camera position and orientation uncertainties

	X [m]	Y [m]	Z [m]	Omega [degree]	Phi [degree]	Kappa [degree]
Mean	0.002	0.001	0.002	0.002	0.002	0.001
Sigma	0.001	0.000	0.001	0.000	0.000	0.000

Geolocation Details

Absolute Geolocation Variance

Min Error [m]	Max Error [m]	Geolocation Error X [%]	Geolocation Error Y [%]	Geolocation Error Z [%]
-	-0.05	0.00	0.00	0.00
-0.05	-0.04	0.00	0.00	0.00
-0.04	-0.03	0.00	0.00	0.00
-0.03	-0.02	0.00	0.24	0.71
-0.02	-0.01	3.76	8.00	17.41
-0.01	0.00	46.82	40.00	38.59
0.00	0.01	48.24	46.59	16.94
0.01	0.02	1.18	3.76	11.29
0.02	0.03	0.00	1.18	12.71
0.03	0.04	0.00	0.24	1.88
0.04	0.05	0.00	0.00	0.47
0.05	-	0.00	0.00	0.00
**Mean [m]**		-0.000258	-0.000078	0.001895
**Sigma [m]**		0.005134	0.006603	0.012898
**RMS Error [m]**		0.005141	0.006604	0.013036

Min Error and Max Error represent geolocation error intervals between -1.5 and 1.5 times the maximum accuracy of all the images. Columns X, Y, Z show the percentage of images with geolocation errors within the predefined error intervals. The geolocation error is the difference between the initial and computed image positions. Note that the image geolocation errors do not correspond to the accuracy of the observed 3D points.

Relative Geolocation Variance

Relative Geolocation Error	Images X [%]	Images Y [%]	Images Z [%]
[-1.00, 1.00]	99.76	95.53	98.59
[-2.00, 2.00]	100.00	99.76	100.00
[-3.00, 3.00]	100.00	100.00	100.00
**Mean of Geolocation Accuracy [m]**	0.013144	0.013144	0.026475
**Sigma of Geolocation Accuracy [m]**	0.000543	0.000543	0.002846

Images X, Y, Z represent the percentage of images with a relative geolocation error in X, Y, Z.

Geolocation Orientational Variance	RMS [degree]
Omega	0.259
Phi	4.960
Kappa	3.391

Geolocation RMS error of the orientation angles given by the difference between the initial and computed image orientation angles.

Initial Processing Details

System Information

Hardware	CPU: Intel(R) Core(TM) i7-10700 CPU @ 2.90GHz<br>RAM: 64GB<br>GPU: NVIDIA Quadro P2200 (Driver: 31.0.15.1737), Intel(R) UHD Graphics 630 (Driver: 27.20.100.9664)
Operating System	Windows 10 Enterprise, 64-bit

Coordinate Systems

Image Coordinate System	WGS 84
Output Coordinate System	WGS 84 / UTM zone 60S (+25m)

Processing Options

Detected Template	No Template Available
Keypoints Image Scale	Full, Image Scale: 1
Advanced: Matching Image Pairs	Free Flight or Terrestrial
Advanced: Matching Strategy	Use Geometrically Verified Matching: yes
Advanced: Keypoint Extraction	Targeted Number of Keypoints: Automatic
Advanced: Calibration	Calibration Method: Geolocation Based<br>Internal Parameters Optimization: All<br>External Parameters Optimization: All<br>Rematch: Auto, yes

Point Cloud Densification details

Processing Options

Image Scale	multiscale, 1/2 (Half image size, Default)
Point Density	Optimal
Minimum Number of Matches	3
3D Textured Mesh Generation	yes
3D Textured Mesh Settings:	Resolution: High Resolution<br>Color Balancing: no
LOD	Generated: no

Advanced: 3D Textured Mesh Settings	Sample Density Divider: 1
Advanced: Image Groups	group1
Advanced: Use Processing Area	yes
Advanced: Use Annotations	yes
Time for Point Cloud Densification	02h:38m:18s
Time for Point Cloud Classification	NA
Time for 3D Textured Mesh Generation	38m:21s

Results

Number of Generated Tiles	1
Number of 3D Densified Points	17670758
Average Density (per m^3)	192.01

DSM, Orthomosaic and Index Details

Processing Options

DSM and Orthomosaic Resolution	1 x GSD (3.32 [cm/pixel])
DSM Filters	Noise Filtering: yes<br>Surface Smoothing: yes, Type: Sharp
Raster DSM	Generated: yes<br>Method: Inverse Distance Weighting<br>Merge Tiles: yes
Orthomosaic	Generated: yes<br>Merge Tiles: yes<br>GeoTIFF Without Transparency: no<br>Google Maps Tiles and KML: no
Time for DSM Generation	13m:58s
Time for Orthomosaic Generation	46m:23s
Time for DTM Generation	00s
Time for Contour Lines Generation	00s
Time for Reflectance Map Generation	00s
Time for Index Map Generation	00s

Quality Report

Generated with Pix4Dmapper version 4.4.12

Important: Click on the different icons for:

Help to analyze the results in the Quality Report

Additional information about the sections

Click here for additional tips to analyze the Quality Report

Summary

Project	Jam Roll_09112
Processed	2023-11-10 00:05:[illegible]
Camera Model Name(s)	FC6310R_8.8_4864x3648 (RGB)
Average Ground Sampling Distance (GSD)	0.89 cm / 0.35 in
Area Covered	0.040 km^2 / 4.0224 ha / 0.02 sq. mi. / 9.9447 acres
Time for Initial Processing (without report)	35m:58s

Quality Check

**Images**	median of 51631 keypoints per image	✓
**Dataset**	1047 out of 1085 images calibrated (96%), all images enabled, 8 blocks	⚠
**Camera Optimization**	0.89% relative difference between initial and optimized internal camera parameters	✓
**Matching**	median of 4621.19 matches per calibrated image	✓
**Georeferencing**	yes, no 3D GCP	⚠

Preview

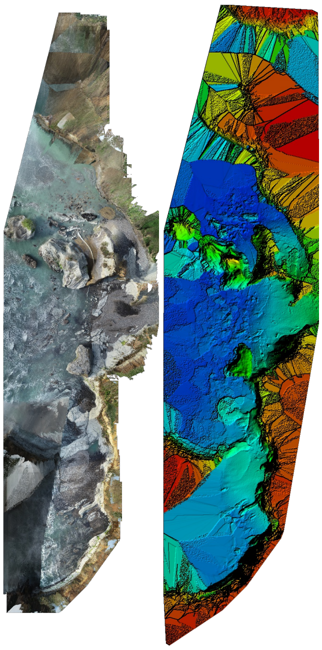

Figure 1: Orthomosaic and the corresponding sparse Digital Surface Model (DSM) before densification.

Calibration Details

Number of Calibrated Images	1047 out of 1085
Number of Geolocated Images	1085 out of 1085

Initial Image Positions

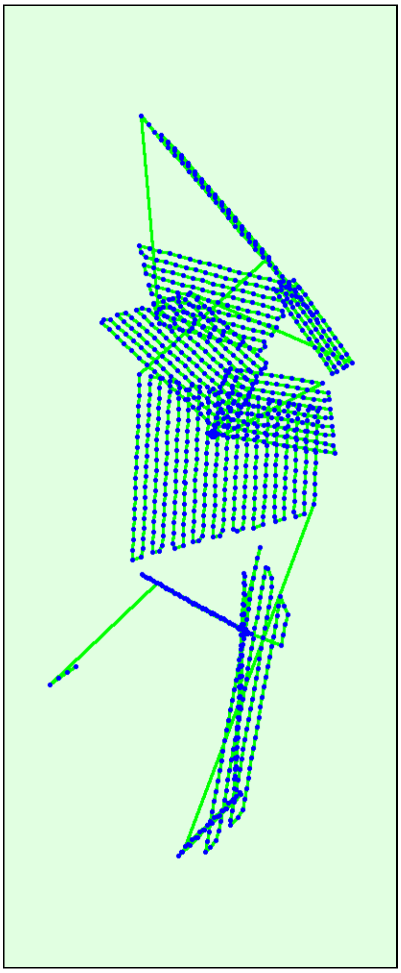

Figure 2: Top view of the initial image position. The green line follows the position of the images in time starting from the large blue dot.

Computed Image/GCPs/Manual Tie Points Positions

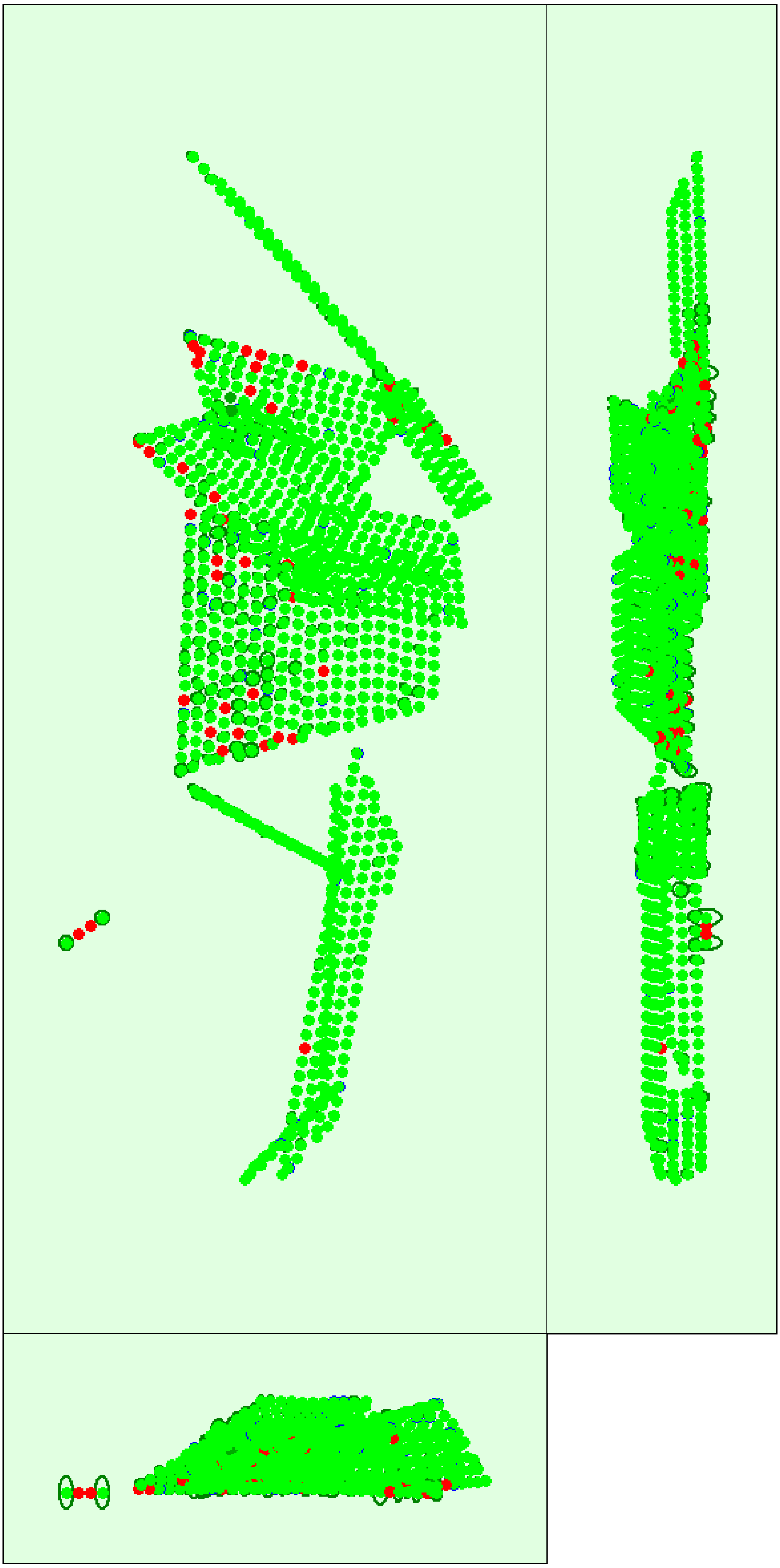

Uncertainty ellipses 500x magnified

Figure 3: Offset between initial (blue dots) and computed (green dots) image positions as well as the offset between the GCPs initial positions (blue crosses) and their computed positions (green crosses) in the top-view (XY plane), front-view (XZ plane), and side-view (YZ plane). Red dots indicate disabled or uncalibrated images. Dark green ellipses indicate the absolute position uncertainty of the bundle block adjustment result.

Absolute camera position and orientation uncertainties

	X [m]	Y [m]	Z [m]	Omega [degree]	Phi [degree]	Kappa [degree]
Mean	0.002	0.002	0.003	0.010	0.006	0.009
Sigma	0.001	0.001	0.001	0.045	0.008	0.020

Overlap

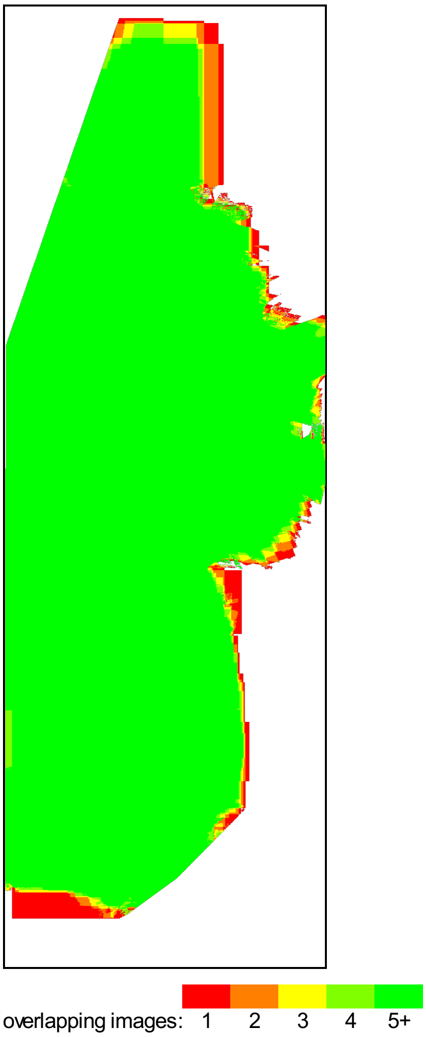

Figure 4: Number of overlapping images computed for each pixel of the orthomosaic.
Red and yellow areas indicate low overlap for which poor results may be generated. Green areas indicate an overlap of over 5 images for every pixel. Good quality results will be generated as long as the number of keypoint matches is also sufficient for these areas (see Figure 5 for keypoint matches).

Bundle Block Adjustment Details

Number of 2D Keypoint Observations for Bundle Block Adjustment	7371570
Number of 3D Points for Bundle Block Adjustment	2746293
Mean Reprojection Error [pixels]	0.094

Internal Camera Parameters

FC6310R_8.8_4864x3648 (RGB). Sensor Dimensions: 11.407 [mm] x 8.556 [mm]

EXIF ID: FC6310R_8.8_4864x3648

	Focal Length	Principal Point x	Principal Point y	R1	R2	R3	T1	T2

Initial Values	3666.840 [pixel]<br>8.600 [mm]	2420.300 [pixel]<br>5.676 [mm]	1835.990 [pixel]<br>4.306 [mm]	-0.270	0.112	-0.032	0.000	-0.001
Optimized Values	3633.933 [pixel]<br>8.523 [mm]	2425.144 [pixel]<br>5.688 [mm]	1838.803 [pixel]<br>4.312 [mm]	-0.269	0.112	-0.034	-0.000	-0.001
Uncertainties (Sigma)	0.062 [pixel]<br>0.000 [mm]	0.079 [pixel]<br>0.000 [mm]	0.071 [pixel]<br>0.000 [mm]	0.000	0.000	0.000	0.000	0.000

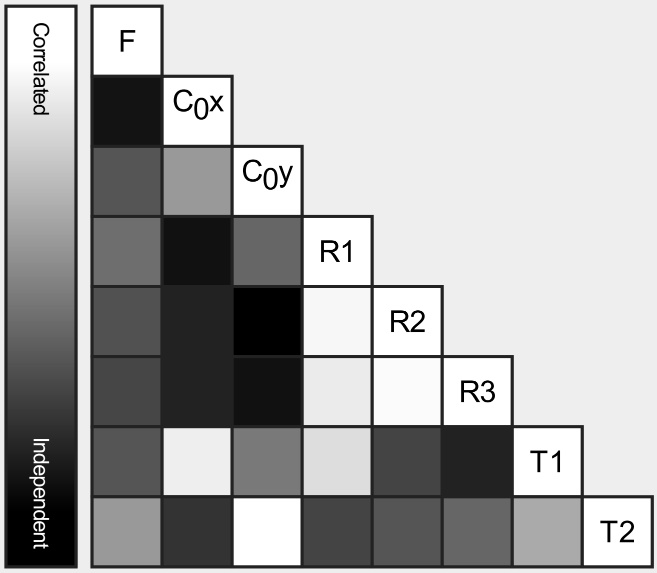

The correlation between camera internal parameters determined by the bundle adjustment. White indicates a full correlation between the parameters, ie. any change in one can be fully compensated by the other. Black indicates that the parameter is completely independent, and is not affected by other parameters.

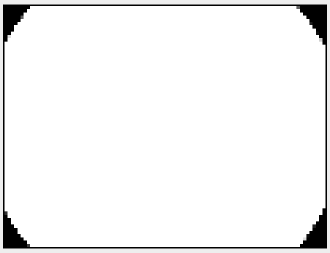

The number of Automatic Tie Points (ATPs) per pixel, averaged over all images of the camera model, is color coded between black and white. White indicates that, on average, more than 16 ATPs have been extracted at the pixel location. Black indicates that, on average, 0 ATPs have been extracted at the pixel location. Click on the image to the see the average direction and magnitude of the re-projection error for each pixel. Note that the vectors are scaled for better visualization. The scale bar indicates the magnitude of 1 pixel error.

2D Keypoints Table

	Number of 2D Keypoints per Image	Number of Matched 2D Keypoints per Image
Median	51631	4621
Min	19759	18
Max	78380	38854
Mean	50349	7041

3D Points from 2D Keypoint Matches

	Number of 3D Points Observed
In 2 Images	1796827
In 3 Images	531790
In 4 Images	209660
In 5 Images	95652
In 6 Images	48091
In 7 Images	25367
In 8 Images	13707
In 9 Images	8234
In 10 Images	5185
In 11 Images	3161
In 12 Images	2283
In 13 Images	1574
In 14 Images	1093
In 15 Images	838
In 16 Images	662

In 17 Images	485
In 18 Images	419
In 19 Images	341
In 20 Images	271
In 21 Images	203
In 22 Images	141
In 23 Images	94
In 24 Images	81
In 25 Images	38
In 26 Images	30
In 27 Images	15
In 28 Images	18
In 29 Images	11
In 30 Images	4
In 31 Images	5
In 32 Images	4
In 33 Images	2
In 34 Images	5
In 35 Images	1
In 37 Images	1

2D Keypoint Matches

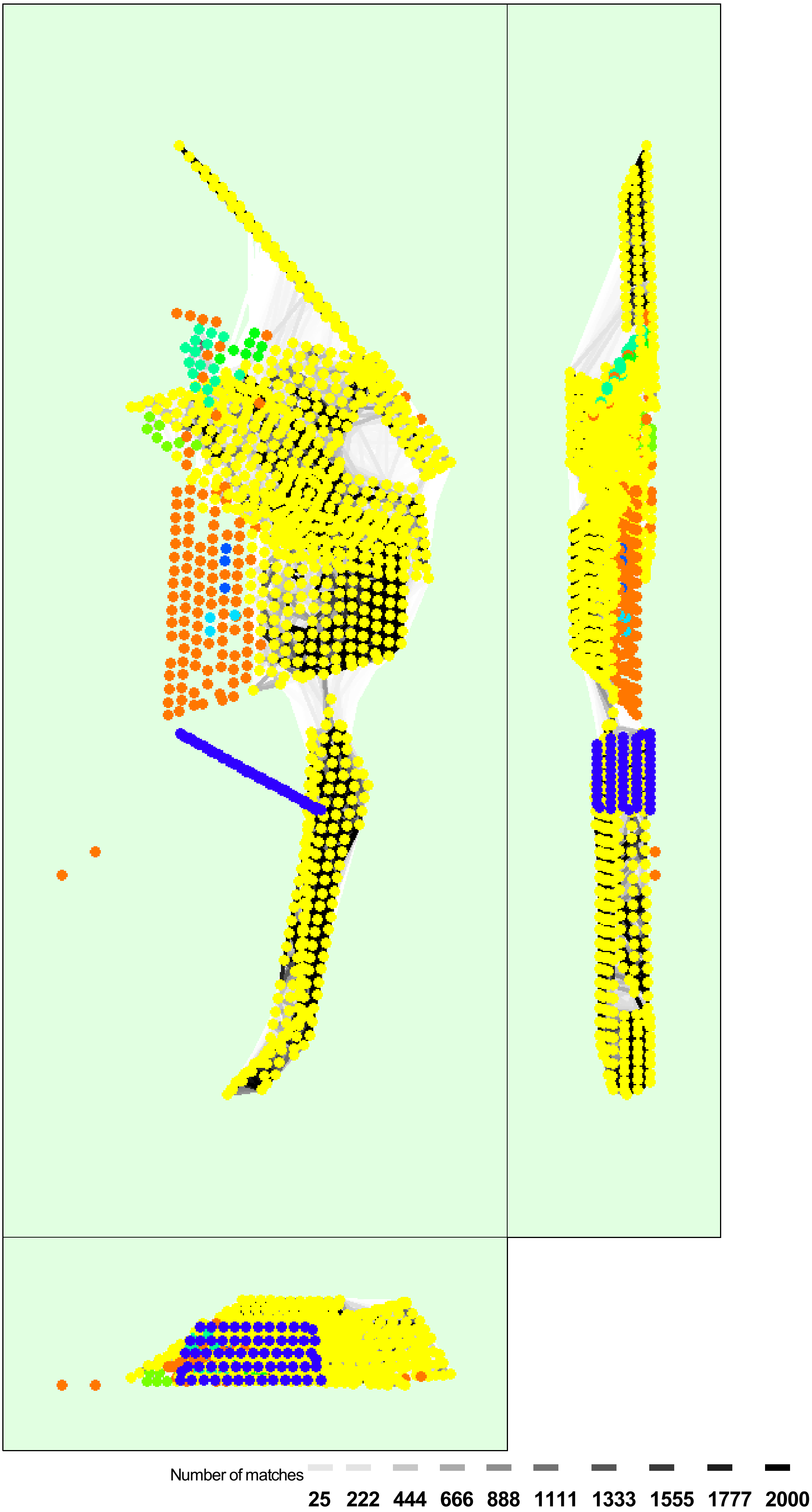

Figure 5: Computed image positions with links between matched images. The darkness of the links indicates the number of matched 2D keypoints between the images. Bright links indicate weak links and require manual tie points or more images.

Geolocation Details

Absolute Geolocation Variance

Min Error [m]	Max Error [m]	Geolocation Error X [%]	Geolocation Error Y [%]	Geolocation Error Z [%]
-	-0.05	0.29	1.05	3.35
-0.05	-0.04	0.67	1.25	1.44
-0.04	-0.03	1.15	0.48	1.92
-0.03	-0.02	2.30	1.92	4.41
-0.02	-0.01	7.85	5.08	10.63
-0.01	0.00	41.19	38.31	23.47
0.00	0.01	34.96	39.75	31.51
0.01	0.02	6.23	8.43	12.93
0.02	0.03	2.78	2.01	4.60
0.03	0.04	1.34	0.48	0.96
0.04	0.05	0.96	0.57	1.25
0.05	-	0.29	0.67	3.54
**Mean [m]**		-0.000098	-0.000117	0.000285
**Sigma [m]**		0.013437	0.014462	0.030920
**RMS Error [m]**		0.013437	0.014463	0.030921

Min Error and Max Error represent geolocation error intervals between -1.5 and 1.5 times the maximum accuracy of all the images. Columns X, Y, Z show the percentage of images with geolocation errors within the predefined error intervals. The geolocation error is the difference between the initial and computed image positions. Note that the image geolocation errors do not correspond to the accuracy of the observed 3D points.

Relative Geolocation Variance

Relative Geolocation Error	Images X [%]	Images Y [%]	Images Z [%]
[-1.00, 1.00]	77.49	80.46	81.42
[-2.00, 2.00]	91.19	92.72	91.48
[-3.00, 3.00]	96.36	95.79	94.54
**Mean of Geolocation Accuracy [m]**	0.011343	0.011343	0.023511
**Sigma of Geolocation Accuracy [m]**	0.000702	0.000702	0.001671

Images X, Y, Z represent the percentage of images with a relative geolocation error in X, Y, Z.

Geolocation Orientational Variance	RMS [degree]
Omega	12.167
Phi	2.302
Kappa	13.533

Geolocation RMS error of the orientation angles given by the difference between the initial and computed image orientation angles.

Initial Processing Details

System Information

Hardware	CPU: Intel(R) Core(TM) i7-10700 CPU @ 2.90GHz<br>RAM: 64GB<br>GPU: unknown graphics card (Driver: unknown)
Operating System	Windows 10 Enterprise, 64-bit

Coordinate Systems

Image Coordinate System	WGS 84
Output Coordinate System	WGS 84 / UTM zone 60S (+25m)

Processing Options

Detected Template	No Template Available
Keypoints Image Scale	Full, Image Scale: 1
Advanced: Matching Image Pairs	Aerial Grid or Corridor
Advanced: Matching Strategy	Use Geometrically Verified Matching: yes
Advanced: Keypoint Extraction	Targeted Number of Keypoints: Automatic
Advanced: Calibration	Calibration Method: Geolocation Based<br>Internal Parameters Optimization: All<br>External Parameters Optimization: All<br>Rematch: Auto, no

Point Cloud Densification details

Processing Options

Image Scale	multiscale, 1/2 (Half image size, Default)
Point Density	Optimal
Minimum Number of Matches	3
3D Textured Mesh Generation	yes
3D Textured Mesh Settings:	Resolution: High Resolution<br>Color Balancing: no
LOD	Generated: no
Advanced: 3D Textured Mesh Settings	Sample Density Divider: 1
Advanced: Image Groups	group1
Advanced: Use Processing Area	yes
Advanced: Use Annotations	yes
Time for Point Cloud Densification	02h:23m:24s
Time for Point Cloud Classification	01m:42s
Time for 3D Textured Mesh Generation	55m:45s

Results

Number of Generated Tiles	3
Number of 3D Densified Points	51930138
Average Density (per m^3)	2736.68

Quality Report

Generated with Pix4Dmapper version 4.4.12

Important: Click on the different icons for:

Help to analyze the results in the Quality Report

Additional information about the sections

Click here for additional tips to analyze the Quality Report

Summary

Project	Tt River 2910
Processed	2023-11-06 20:41:35
Camera Model Name(s)	FC6310R_8.8_4864x3648 (RGB)
Average Ground Sampling Distance (GSD)	1.05 cm / 0.41 in
Area Covered	0.079 km^2 / 7.8912 ha / 0.03 sq. mi. / 19.5096 acres
Time for Initial Processing (without report)	33m:40s

Quality Check

**Images**	median of 52863 keypoints per image	✓
**Dataset**	486 out of 486 images calibrated (100%), all images enabled	✓
**Camera Optimization**	0.97% relative difference between initial and optimized internal camera parameters	✓
**Matching**	median of 12258.2 matches per calibrated image	✓
**Georeferencing**	yes, no 3D GCP	⚠

Preview

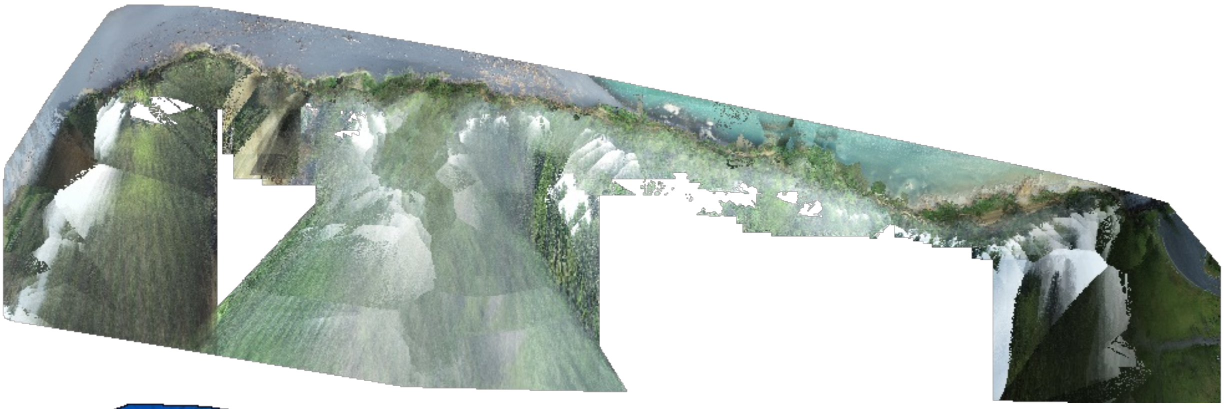

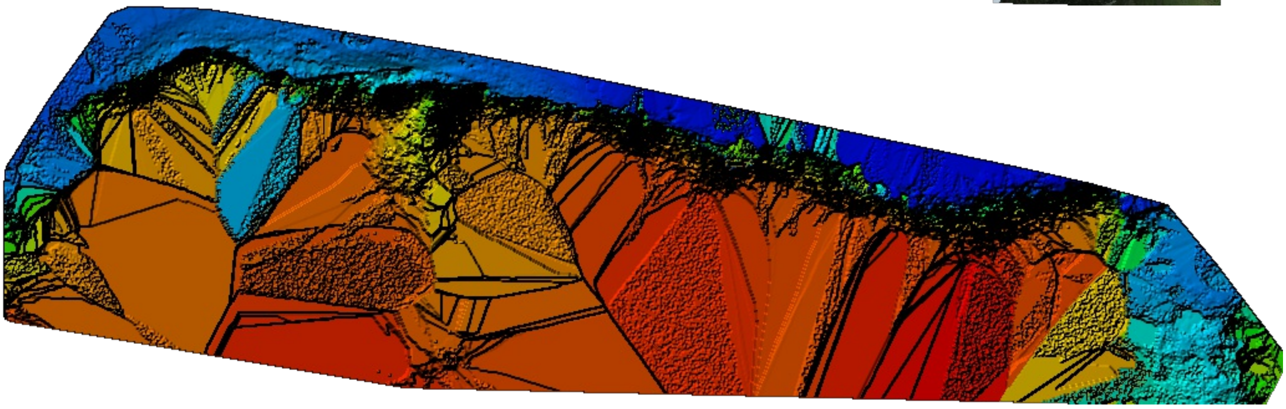

Figure 1: Orthomosaic and the corresponding sparse Digital Surface Model (DSM) before densification.

Calibration Details

Number of Calibrated Images	486 out of 486
Number of Geolocated Images	486 out of 486

Initial Image Positions

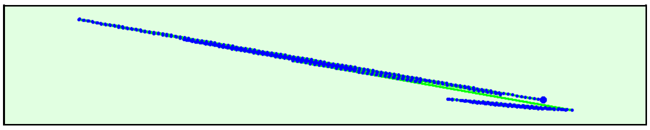

Figure 2: Top view of the initial image position. The green line follows the position of the images in time starting from the large blue dot.

Computed Image/GCPs/Manual Tie Points Positions

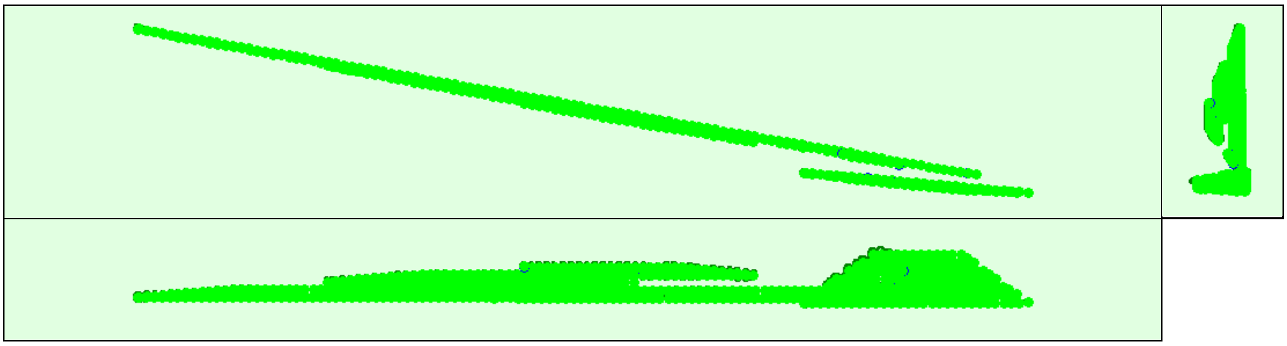

Uncertainty ellipses 1000x magnified

Figure 3: Offset between initial (blue dots) and computed (green dots) image positions as well as the offset between the GCPs initial positions (blue crosses) and their computed positions (green crosses) in the top-view (XY plane), front-view (XZ plane), and side-view (YZ plane). Dark green ellipses indicate the absolute position uncertainty of the bundle block adjustment result.

Absolute camera position and orientation uncertainties

	X [m]	Y [m]	Z [m]	Omega [degree]	Phi [degree]	Kappa [degree]
Mean	0.002	0.001	0.002	0.004	0.003	0.002
Sigma	0.000	0.000	0.000	0.001	0.000	0.001

Overlap

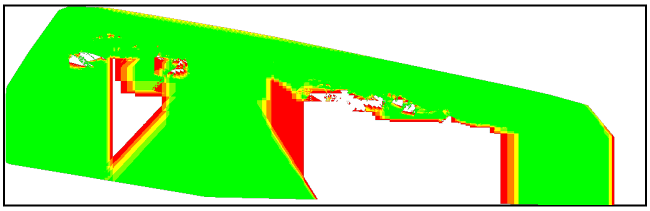

Number of overlapping images: 1 2 3 4 5+

Figure 4: Number of overlapping images computed for each pixel of the orthomosaic.
Red and yellow areas indicate low overlap for which poor results may be generated. Green areas indicate an overlap of over 5 images for every pixel. Good quality results will be generated as long as the number of keypoint matches is also sufficient for these areas (see Figure 5 for keypoint matches).

Bundle Block Adjustment Details

Number of 2D Keypoint Observations for Bundle Block Adjustment	5991970
Number of 3D Points for Bundle Block Adjustment	2033549
Mean Reprojection Error [pixels]	0.166

Internal Camera Parameters

FC6310R_8.8_4864x3648 (RGB). Sensor Dimensions: 11.407 [mm] x 8.556 [mm]

EXIF ID: FC6310R_8.8_4864x3648

	Focal Length	Principal Point x	Principal Point y	R1	R2	R3	T1	T2
Initial Values	3666.840 [pixel]<br>8.600 [mm]	2420.300 [pixel]<br>5.676 [mm]	1835.990 [pixel]<br>4.306 [mm]	-0.270	0.112	-0.032	0.000	-0.001
Optimized Values	3631.200 [pixel]<br>8.516 [mm]	2419.739 [pixel]<br>5.675 [mm]	1838.516 [pixel]<br>4.312 [mm]	-0.269	0.114	-0.036	-0.000	-0.001
Uncertainties (Sigma)	0.171 [pixel]<br>0.000 [mm]	0.153 [pixel]<br>0.000 [mm]	0.151 [pixel]<br>0.000 [mm]	0.000	0.000	0.000	0.000	0.000

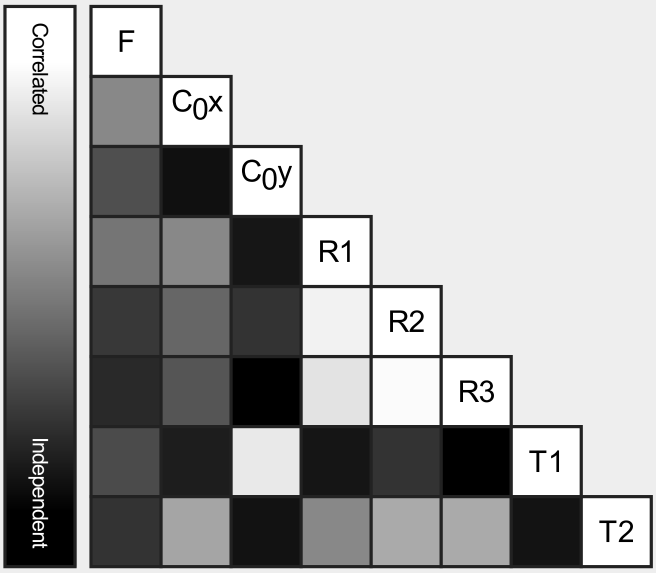

The correlation between camera internal parameters determined by the bundle adjustment. White indicates a full correlation between the parameters, ie. any change in one can be fully compensated by the other. Black indicates that the parameter is completely independent, and is not affected by other parameters.

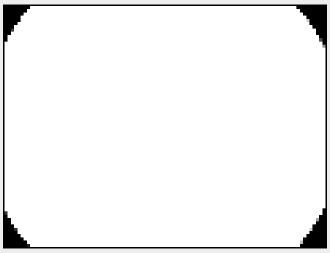

The number of Automatic Tie Points (ATPs) per pixel, averaged over all images of the camera model, is color coded between black and white. White indicates that, on average, more than 16 ATPs have been extracted at the pixel location. Black indicates that, on average, 0 ATPs have been extracted at the pixel location. Click on the image to the see the average direction and magnitude of the re-projection error for each pixel. Note that the vectors are scaled for better visualization. The scale bar indicates the magnitude of 1 pixel error.

2D Keypoints Table

	Number of 2D Keypoints per Image	Number of Matched 2D Keypoints per Image
Median	52863	12258
Min	19951	1703
Max	79965	34601
Mean	50708	12329

3D Points from 2D Keypoint Matches

	Number of 3D Points Observed
In 2 Images	1420271

In 3 Images	295682
In 4 Images	115642
In 5 Images	58928
In 6 Images	35243
In 7 Images	23466
In 8 Images	16626
In 9 Images	12217
In 10 Images	9491
In 11 Images	7307
In 12 Images	5962
In 13 Images	4908
In 14 Images	4065
In 15 Images	3326
In 16 Images	2839
In 17 Images	2410
In 18 Images	2119
In 19 Images	1810
In 20 Images	1529
In 21 Images	1318
In 22 Images	1068
In 23 Images	911
In 24 Images	762
In 25 Images	667
In 26 Images	522
In 27 Images	466
In 28 Images	425
In 29 Images	416
In 30 Images	337
In 31 Images	281
In 32 Images	256
In 33 Images	234
In 34 Images	191
In 35 Images	181
In 36 Images	162
In 37 Images	152
In 38 Images	141
In 39 Images	119
In 40 Images	101
In 41 Images	85
In 42 Images	83
In 43 Images	77
In 44 Images	71
In 45 Images	57
In 46 Images	64
In 47 Images	44
In 48 Images	50
In 49 Images	42
In 50 Images	35
In 51 Images	42
In 52 Images	31
In 53 Images	28
In 54 Images	18
In 55 Images	18
In 56 Images	19
In 57 Images	22
In 58 Images	19
In 59 Images	11
In 60 Images	10
In 61 Images	16

In 62 Images	17
In 63 Images	14
In 64 Images	6
In 65 Images	9
In 66 Images	12
In 67 Images	7
In 68 Images	3
In 69 Images	9
In 70 Images	6
In 71 Images	5
In 72 Images	8
In 73 Images	5
In 74 Images	4
In 75 Images	4
In 76 Images	2
In 77 Images	12
In 78 Images	2
In 79 Images	1
In 80 Images	3
In 81 Images	2
In 82 Images	3
In 83 Images	7
In 84 Images	1
In 85 Images	1
In 87 Images	1
In 88 Images	2
In 89 Images	2
In 92 Images	1
In 97 Images	1
In 98 Images	1
In 100 Images	2
In 102 Images	1
In 103 Images	1
In 104 Images	1

2D Keypoint Matches

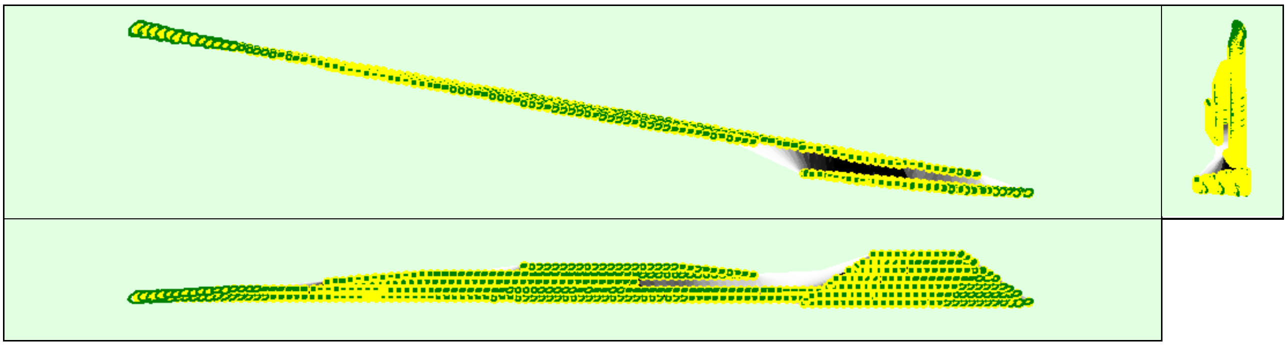

Uncertainty ellipses 100x magnified

Number of matches

25 222 444 666 888 1111 1333 1555 1777 2000

Figure 5: Computed image positions with links between matched images. The darkness of the links indicates the number of matched 2D keypoints between the images. Bright links indicate weak links and require manual tie points or more images. Dark green ellipses indicate the relative camera position uncertainty of the bundle block adjustment result.

Relative camera position and orientation uncertainties

	X[m]	Y[m]	Z[m]	Omega [degree]	Phi [degree]	Kappa [degree]
Mean	0.015	0.013	0.010	0.013	0.014	0.007

Sigma	0.009	0.008	0.006	0.001	0.005	0.003

Geolocation Details

Absolute Geolocation Variance

Min Error [m]	Max Error [m]	Geolocation Error X [%]	Geolocation Error Y [%]	Geolocation Error Z [%]
-	-0.05	0.00	0.00	0.21
-0.05	-0.04	0.00	0.00	0.62
-0.04	-0.03	0.00	0.00	1.03
-0.03	-0.02	0.41	0.00	1.65
-0.02	-0.01	3.29	2.26	13.58
-0.01	0.00	44.03	46.09	33.54
0.00	0.01	50.00	50.00	33.54
0.01	0.02	2.26	1.65	10.91
0.02	0.03	0.00	0.00	3.29
0.03	0.04	0.00	0.00	1.03
0.04	0.05	0.00	0.00	0.62
0.05	-	0.00	0.00	0.00
**Mean [m]**		-0.000055	-0.000010	0.000100
**Sigma [m]**		0.005278	0.004677	0.012845
**RMS Error [m]**		0.005279	0.004677	0.012846

Min Error and Max Error represent geolocation error intervals between -1.5 and 1.5 times the maximum accuracy of all the images. Columns X, Y, Z show the percentage of images with geolocation errors within the predefined error intervals. The geolocation error is the difference between the initial and computed image positions. Note that the image geolocation errors do not correspond to the accuracy of the observed 3D points.

Relative Geolocation Variance

Relative Geolocation Error	Images X [%]	Images Y [%]	Images Z [%]
[-1.00, 1.00]	92.80	94.86	91.77
[-2.00, 2.00]	99.59	100.00	98.56
[-3.00, 3.00]	100.00	100.00	100.00
**Mean of Geolocation Accuracy [m]**	0.009803	0.009803	0.021096
**Sigma of Geolocation Accuracy [m]**	0.000542	0.000542	0.001291

Images X, Y, Z represent the percentage of images with a relative geolocation error in X, Y, Z.

Geolocation Orientational Variance	RMS [degree]
Omega	0.871
Phi	7.091
Kappa	3.142

Geolocation RMS error of the orientation angles given by the difference between the initial and computed image orientation angles.

Initial Processing Details

System Information

Hardware	CPU: Intel(R) Core(TM) i7-10700 CPU @ 2.90GHz<br>RAM: 64GB<br>GPU: unknown graphics card (Driver: unknown)

Operating System	Windows 10 Enterprise, 64-bit

Coordinate Systems

Image Coordinate System	WGS 84
Output Coordinate System	WGS 84 / UTM zone 60S (+25m)

Processing Options

Detected Template	No Template Available
Keypoints Image Scale	Full, Image Scale: 1
Advanced: Matching Image Pairs	Free Flight or Terrestrial
Advanced: Matching Strategy	Use Geometrically Verified Matching: yes
Advanced: Keypoint Extraction	Targeted Number of Keypoints: Automatic
Advanced: Calibration	Calibration Method: Geolocation Based<br>Internal Parameters Optimization: All<br>External Parameters Optimization: All<br>Rematch: Auto, yes

Point Cloud Densification details

Processing Options

Image Scale	multiscale, 1/2 (Half image size, Default)
Point Density	Optimal
Minimum Number of Matches	3
3D Textured Mesh Generation	yes
3D Textured Mesh Settings:	Resolution: High Resolution<br>Color Balancing: no
LOD	Generated: no
Advanced: 3D Textured Mesh Settings	Sample Density Divider: 1
Advanced: Image Groups	group1
Advanced: Use Processing Area	yes
Advanced: Use Annotations	yes
Time for Point Cloud Densification	01h:33m:19s
Time for Point Cloud Classification	NA
Time for 3D Textured Mesh Generation	33m:24s

Results

Number of Generated Tiles	3
Number of 3D Densified Points	39977457
Average Density (per m^3)	1790.73
